# Interactions between natural products and cancer treatments: underlying mechanisms and clinical importance

**DOI:** 10.1007/s00280-023-04504-z

**Published:** 2023-01-27

**Authors:** Wai-Jo Jocelin Chan, Jeffry Adiwidjaja, Andrew J. McLachlan, Alan V. Boddy, Joanna E. Harnett

**Affiliations:** 1 Sydney Pharmacy School, Faculty of Medicine and Health, The University of Sydney, Sydney, NSW 2006 Australia; 2 Division of Pharmacotherapy and Experimental Therapeutics, UNC Eshelman School of Pharmacy, University of North Carolina, Chapel Hill, NC 27599 USA; 3 Clinical and Health Sciences, University of South Australia, Adelaide, SA 5001 Australia

**Keywords:** Natural products, Interactions, Cancer, Complementary medicines, Herbal medicines, Chemotherapy

## Abstract

Natural products, also referred to as dietary supplements, complementary and alternative medicines, and health or food supplements are widely used by people living with cancer. These products are predominantly self-selected and taken concurrently with cancer treatments with the intention of improving quality of life, immune function and reducing cancer symptoms and treatment side effects. Concerns have been raised that concurrent use may lead to interactions resulting in adverse effects and unintended treatment outcomes. This review provides an overview of the mechanisms by which these interactions can occur and the current evidence about specific clinically important natural product–drug interactions. Clinical studies investigating pharmacokinetic interactions provide evidence that negative treatment outcomes may occur when *Hypericum perforatum*, Grapefruit, *Schisandra sphenanthera, Curcuma longa* or *Hydrastis canadensis* are taken concurrently with common cancer treatments. Conversely, pharmacodynamic interactions between Hangeshashinto (TJ-14) and some cancer treatments have been shown to reduce the side effects of diarrhoea and oral mucositis. In summary, research in this area is limited and requires further investigation.

**Supplementary Information:**

The online version contains supplementary material available at 10.1007/s00280-023-04504-z.

## Introduction

The National Center for Complementary and Integrative Health (NCCIH) currently classify products containing herbs, vitamins and minerals, prebiotics and probiotics as ‘natural products (NPs)’ [1]. NP use is prevalent throughout the world, including by people living with cancer. A cross-sectional study (*n* = 128) revealed people living with cancer used NPs more than people with acute and chronic non-malignant diseases, and only 50% had disclosed their NP use to their physicians [2]. An Australian study reported that 65% (*n* = 381) of people living with cancer had utilised at least one form of complementary and alternative medicine [3]. Patients perceived benefits associated with NP use include improved quality of life, reduced symptoms and side effects, and improved immune function [3].

A significant percentage of people living with cancer do take NPs and anti-cancer drugs concurrently [4, 5] with almost 35% of people with advanced cancer enrolled into phase I chemotherapy trials (*n* = 212) reporting concurrent use [4]. Another US study estimated 30% (*n* = 820) of cancer patients had used one or more CAM during active cancer treatment [5]. Such prevalent concurrent use of NPs with anti-cancer treatments raises questions and concerns about the potential for interactions that alter the efficacy and safety profiles of cancer treatments. This narrative review aims to describe a selection of clinically important interactions between NPs commonly used in the community and anti-cancer drugs, including (1) cytotoxic drugs with a relatively narrow therapeutic index and (2) small molecule targeted therapy (kinase inhibitors), which are administered daily over a longer treatment period.

## The evidence base for drug–herb and drug–nutrient interactions

**Fig. 1:**
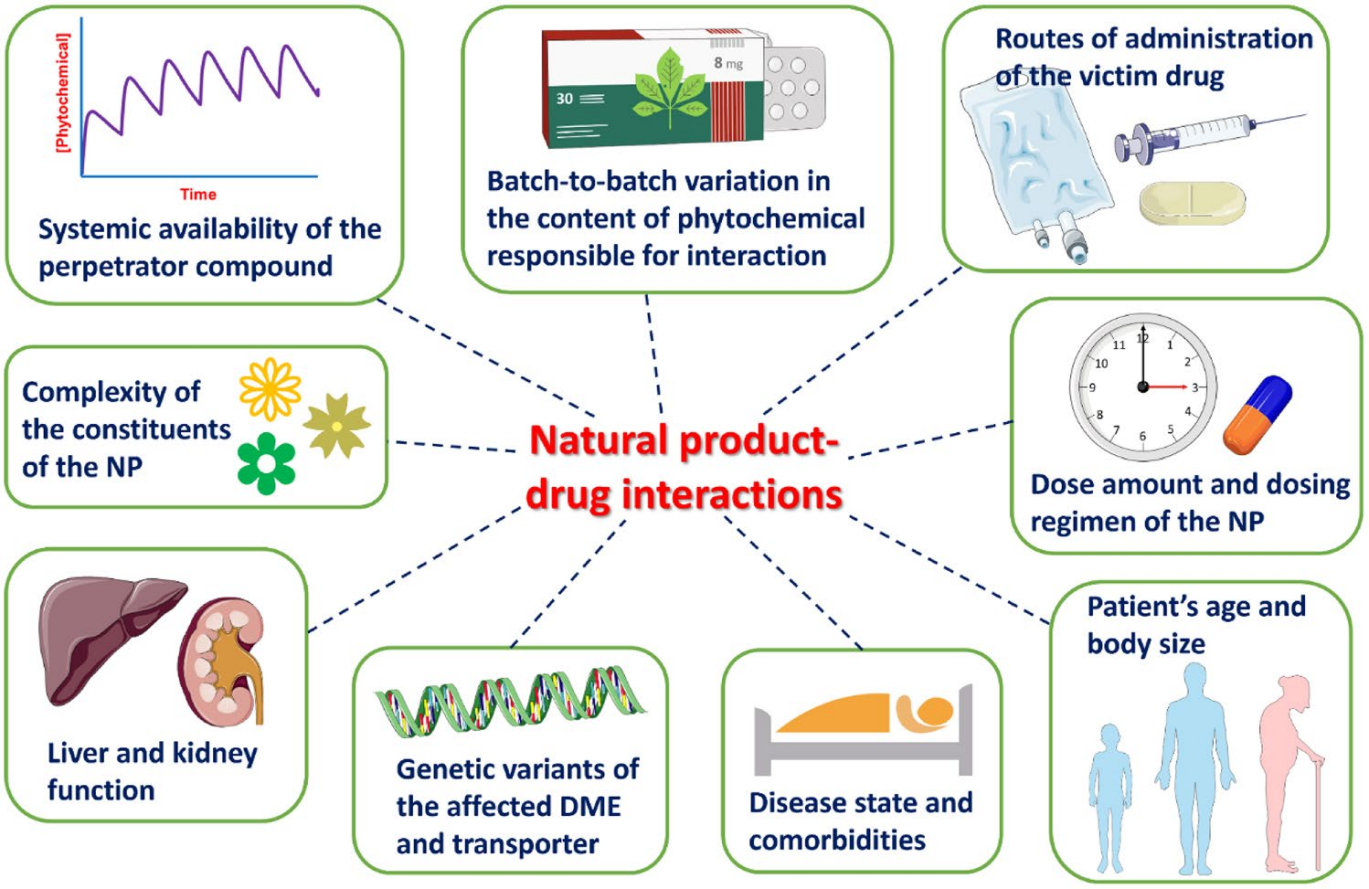
Potential sources of inter-individual variability in the extent of natural product–drug interactions. Freely available medical illustrations from the Servier Medical Art (SMART, https://smart.servier.com) and default illustrations within the Microsoft 365 were used to create the figure. *DME* drug-metabolising enzyme, *NP* natural product

One of the challenges in translating the evidence about interactions between specific NPs (herbs and nutrients) and chemotherapeutic medicines is the wide variation in NP formulations and doses used in studies; and variations in the NP formulations available and accessed by the public [6]. The issue with product variation is highlighted by an Australian study that identified substantial variations between formulations and doses of commonly purchased garlic products [6]. This study also highlighted the common practise of “evidence-borrowing”—using the same evidence to support the use of a herbal medicine across a range of formulations and doses, despite important variation and important differences in the clinical pharmacology of these herbal products [7]. Further, batch-to-batch variations in the phytochemical profiles of plant-based products, including herbal medicines, can occur depending on cultivation and manufacturing practises such as the part of plant used, geographical origin, climate, developmental stage of plant, season/harvesting time, processing and storage [8]. An isolated herbal constituent used in an interaction study is likely to produce different results to that of single herb containing multiple phytoconstituents in regards to drug bioavailability, cumulative or additive pharmacological effects [9]. Physiological characteristics and interacting factors that may explain variability in the extent of interactions between NPs and anti-cancer drugs are outlined in Fig. 1.

The hierarchy of evidence is also another important factor in translating research results into clinically relevant information to guide decision making in healthcare. Whilst there are many in vitro and animal studies investigating interactions in the literature, higher quality of evidence from well-designed controlled clinical trials are more generalisable to people living with cancer [10]. The limitation of in vitro studies using isolated human tissues is omitting important information about the bioavailability and metabolism of phytoconstituents in NPs. The differences between species and their metabolism of drugs and phytochemicals need to be carefully considered and limits the clinical relevance of using animal models for evaluation of NP–drug interactions.

## Mechanism of interactions

The main mechanisms of drug interactions, including those between NP and anti-cancer drugs, are classified as pharmacokinetic, pharmacodynamic and physiochemical [11]. Pharmacokinetic interactions result in the alteration of drug absorption, distribution, metabolism or elimination [11]. The mechanism of pharmacokinetic interactions includes reversible and irreversible inhibition and induction of drug-metabolising enzymes and drug transporters [11]. Pharmacodynamic interactions are the result of pharmacological effects of the affected drugs being altered by the concurrent intake of a NP [11]. Pharmacodynamic interactions can be classified as synergistic, additive or antagonistic [11]. Physiochemical drug interactions occur when the combination of two drugs (including NPs) are incompatible. Such interactions result in the alteration of the physical properties of the drug including its solubility and/or stability which ultimately affect bioavailability of drugs [12]. Most physiochemical interactions are evaluated by in vitro experiments, are largely predictable, and are not commonly evaluated or reported in clinical studies.

**Fig. 2:**
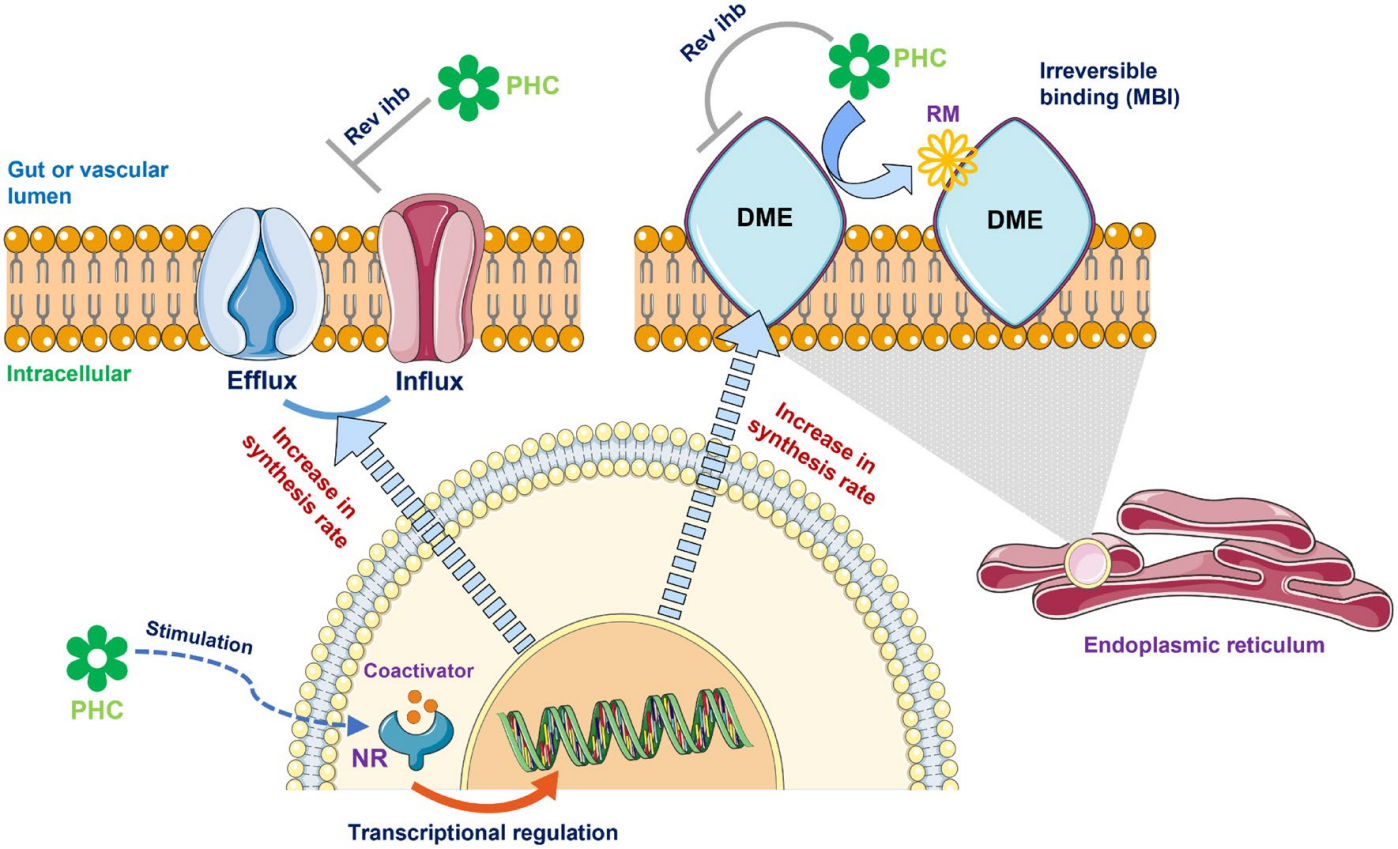
A schematic representation of the proposed mechanisms underlying pharmacokinetic-based natural product–drug interactions. The illustrations were taken from the Servier Medical Art (SMART, https://smart.servier.com). *DME* drug-metabolising enzyme, *MBI* mechanism-based inhibition, *NR* nuclear receptor, *PHC* phytochemical, *rev ihb* reversible inhibition, *RM* reactive metabolite

This review has focussed on the literature reporting pharmacokinetic and pharmacodynamic interactions with the major focus being on the evidence obtained from clinical studies exploring drug–NP interactions reported in people living with cancer. Several key mechanisms underlying the effect of phytoconstituents in NPs on pharmacokinetics of anti-cancer drugs have been proposed, as summarised in Fig. 2.

## Pharmacokinetic interactions

Drug-metabolising enzymes play an important role in drug activation and degradation. They are classified into phase I and II enzymes [13]. Phase I enzymes such as cytochrome P450 (CYP) expressed in the liver, small intestine, kidney and lung are responsible for activation and detoxification of a range of anti-cancer agents [13]. Phase II enzymes including uridine 5′-diphospho-glucuronosyltransferases (UGT), glutathione S-transferases (GST), sulfotransferases (SULT) and N-acetyltransferases catalyse the conjugation of hydrophilic functional groups to increase water solubility and enhance elimination [13]. Induction of drug-metabolising enzymes by NPs may lead to sub-therapeutic drug concentrations, and hence higher risk for therapeutic failure, except for prodrugs where the active metabolite is formed through the affected pathway. Conversely, inhibitory effect of NPs on the metabolising enzymes primarily responsible for elimination of the affected drugs may lead to supratherapeutic drug concentrations, and potentially increases the risk for drug-related adverse effects or toxicity [13]. These interactions are of particular concern for cytotoxic drugs with relatively narrow therapeutic index. The adverse effects of relatively novel targeted therapies differ from those of cytotoxic anti-cancer drugs with respect to frequency and types, due to a more selective mechanism of action [14]. Nevertheless, since targeted therapies are mostly administered orally daily over a long period of treatment, prevention and management of NP–drug interactions with this class of drugs are of importance to improve patients’ quality of life and reduce treatment discontinuation or nonadherence.

NP–drug interactions may also arise from modulation of protein level and activity of drug transporters by phytochemicals present in the NP. Drug transporters are responsible for the delivery of drugs into various tissues and are often a key determinant of the pharmacokinetics of a drug [15]. Drug transporters are expressed in tissues including the intestinal epithelial cells, kidney, hepatocytes, and brain capillary endothelial cells [15]. The most extensively studied drug transporter is P-glycoprotein or ABCB1, an adenosine triphosphate (ATP)-binding cassette (ABC) efflux transporter [16]. ABCB1 regulates the uptake of drugs from the intestinal lumen into enterocytes, from blood circulation into the brain, and enhances the elimination of drugs via hepatic and renal excretion [16]. Overexpression of ABCB1 in cancer cells could potentially result in multi-drug resistance tumours [16]. A substantial number of anti-cancer agents are substrates for ABC transporters, including anthracyclines, vinca alkaloids, topoisomerase I inhibitors, docetaxel, paclitaxel, tamoxifen and imatinib [17]. Induction or inhibition of drug transporters such as ABCB1 could potentially alter the pharmacokinetics of anti-cancer agents [16]. Inhibition of organic anion transporting polypeptide (OATP), e.g. hepatic OATP1B1 and intestinal OATP2B1, by constituents of NPs (including NPs found in juices) has also been proposed as a plausible mechanism underlying the interactions with drug substrates of the transporters [18, 19]. However, the clinical importance of OATP-mediated interactions between NPs and anti-cancer drugs is still largely unexplored. Whilst clinically relevant induction of the ABC efflux transporters by phytochemicals (Fig. 2) has been recognised, e.g. induction of P-glycoprotein by hyperforin contained in St John’s wort extract [20], available clinical evidence and in vitro data has been conflicting as to whether influx transporters (OATP) are inducible by drugs or plant-derived compounds [21].

Inhibition and induction of drug-metabolising enzymes and/or transporters represent mechanistically distinct processes [22], as outlined in Fig. 2. Induction of drug-metabolising enzymes and transporters is an indirect process involving binding and stimulation of nuclear receptors (e.g. pregnane X receptor [PXR]) [23] that regulate the expression of genes encoding the corresponding proteins. Drug interactions with inducers typically require several days to reach maximum effect, depending on the turnover rates of the corresponding enzymes or transporters, but may persist beyond cessation of the consumption of the perpetrator NPs [22]. Unlike the induction mechanism, reversible inhibition of metabolising enzymes and transporters is typically of rapid onset upon exposure to the perpetrator NP constituent(s). Nevertheless, the extent of NP–drug interactions due to reversible inhibition may be higher following multiple doses of the perpetrator NPs as phytochemicals responsible for the interactions accumulate in the body, attaining significant steady-state concentrations. The extent of interactions related to reversible inhibition may also depend on the interval between the administration of NPs and anti-cancer drugs. For example, delaying the ingestion of co-administered NPs until after the absorption of oral anti-cancer drugs being complete or almost complete (also known as dose staggering) [24] may help minimise the extent of undesirable NP–drug interactions.

Several phytochemicals (e.g. furanocoumarins contained in grapefruit juice) [25] have also demonstrated an irreversible time-dependent inhibitory property toward CYP enzymes, also known as *mechanism-based inhibition*. Interactions with mechanism-based inhibitors arise when the affected enzyme is responsible for the metabolic conversion of the perpetrator compounds into reactive metabolites which can then irreversibly bind and inactivate the enzyme [26]. Hence, biosynthesis of new enzyme is required for recovery of in vivo activity of the affected enzymes [22]. Mechanism-based inhibition is typically observed following multiple dose administration of the NPs and may persist even after the systemic concentration of the perpetrator phytochemicals is relatively low [27].

Several functional groups commonly found in the perpetrator compounds have been associated with higher liability to drug interactions through mechanism-based inhibition. One notable example of the so-called ‘structural alerts’ is the methylenedioxyphenyl functional group which is present in several *Schisandra* lignans and goldenseal alkaloids [28]. Both NPs have been associated with in vitro mechanism-based inhibition of CYP3A enzymes [29, 30] with evidence for clinically significant drug interactions, as highlighted in the next section.

Genetic polymorphism in the modulated enzymes or transporters may confound the magnitude of interactions with NPs. The general trend is that individuals carrying a functional wild-type allele, otherwise known as extensive metaboliser or transporter phenotype, are more susceptible to and subject to a greater extent of inhibition of the corresponding CYP enzyme or drug transporter compared to that of poor metabolisers or poor transporter phenotype. However, the effect of genotypes on the extent of NP–drug interactions has not yet been well characterised as that of drug–drug–gene interactions [31]. The effect of apple juice on OATP2B1-mediated absorption of fexofenadine was more prominent in individuals carrying the *SLCO2B1* c.1457C > T functional wild-type allele compared to carriers of at least one variant allele, with ratio of systemic exposure of the drug (relative to drinking water) of 0.15 and 0.27, respectively [32]. Similarly, the in vivo effect of constituents of orange juice on systemic exposure of montelukast, a drug substrate of OATP2B1, was dependent on *SLCO2B1* (c.935G > A) genotype [33]. Interestingly, oral co-administration of garlic (600 mg daily for 13 days) appeared to decrease the systemic clearance of docetaxel in patients of African American ancestry who were CYP3A5 expressor (genotyped as CYP3A5*1/*1), but not of Caucasian patients carrying the CYP3A5*3/*3 variant allele (non-expressor) [34]. However, it is important to note that this study was not sufficiently powered to clearly discern the effect of *CYP3A5* genotype from inter-individual variability on the extent of interactions due to the low number of subjects. The interplay between genetic polymorphism and NP–drug interactions in cancer treatment merits further evaluation.

## Clinically significant pharmacokinetic interactions

### *Hypericum perforatum* (St John’s wort)

An estimated 10–25% people with cancer experience major depressive disorder or depressive symptoms [35]. St John’s wort extract is a NP commonly self-selected or prescribed in some countries for the management of symptoms of anxiety and depression [36]. One of the key bioactive constituents of St John’s wort, hyperforin is a well-known CYP3A4 and ABCB1 inducer [37]. Therefore, concurrent use of St John’s wort and anti-cancer agents that are substrates of CYP3A4 and/or ABCB1 can result in a clinically significant pharmacokinetic interaction and unwanted therapeutic outcome [37].

#### Irinotecan

Clinically significant interactions between St. John’s wort and irinotecan have been reported [38]. Irinotecan is a topoisomerase I inhibitor [39]. It is metabolised into its active metabolite SN-38 by carboxylesterase [39]. Irinotecan is metabolised by CYP3A4 to form 7-ethyl-10-[4-N-(5-aminopentanoic acid)-1-piperidino]-carbonyl-oxycamptothecin (APC), an inactive metabolite [39]. The concurrent use of 900 mg of St John’s wort per day, with intravenously infused irinotecan 350 mg/m^2^ once every 3 weeks was shown to significantly reduce plasma levels of SN-38 by 42% and reduce the myelosuppressive activity of irinotecan [38]. The authors hypothesised that the interaction was likely due to the CYP3A4 inducing activity of St John’s wort that increased the conversion of irinotecan to APC and other metabolites that compensate for the elimination pathway through the formation of SN-38 [38]. Another plausible explanation for substantial decreases in SN-38 AUC and C_max_ following pre-treatment with St John’s wort extract was the induction of intestinal UGT1A1 by hyperforin [23]. SN-38 glucuronide undergoes biliary secretion and may be converted back to unconjugated SN-38 with the help of ß-glucuronidase produced by the normal intestinal microbiota [40]. The increase in intestinal glucuronidation of SN-38 due to co-administration with St John’s wort extract may reduce the amount of intact SN-38 available for subsequent absorption (enterohepatic recirculation), and hence decrease the systemic exposure to SN-38. It is worth mentioning that the ratio of plasma SN-38 glucuronide to SN-38 seemed to be unaffected by the interaction [38]. However, changes in systemic concentration of SN-38 glucuronide may not always be reflective of the corresponding changes in local intestinal concentration, particularly when the interaction (UGT1A1 induction) was confined to that in enterocytes.

#### Imatinib

Several studies have highlighted that St John’s wort could significantly affect the pharmacokinetics of imatinib, an oral tyrosine kinase inhibitor, in healthy adults [41, 42]. Imatinib is extensively metabolised by CYP3A4 into an *N*-demethylated piperazine derivative, and is a substrate of P-glycoprotein [43]. Smith et al. evaluated the effect St John’s wort on imatinib pharmacokinetics in an open-label crossover study [41]. The co-administration of 900 mg St John’s wort extract with 400 mg imatinib mesylate daily was associated with a significant reduction in imatinib area under the concentration–time curve (AUC_0–∞_), peak plasma concentration (C_max_) and half-life by 32%, 29%, and 21%, respectively [41]. This result suggests that St John’s wort reduces absorption and increases the elimination rate of imatinib [41].

Similarly, Frye et al. investigated the potential effect of St John’s wort on pharmacokinetics of imatinib and *N*-desmethyl-imatinib [42]. The concurrent use of 900 mg St John’s wort extract and 400 mg imatinib resulted in a 30% reduction in imatinib AUC_0–∞_ and a corresponding 43% increase in imatinib apparent clearance [42]. Imatinib C_max_ and half-life were reduced, whilst the C_max_ of the imatinib metabolite, *N*-desmethyl-imatinib, was increased by 13% [42]. The authors suggested that the overall decrease in imatinib systemic exposure was likely due to increased first pass elimination of imatinib and formation clearance of *N*-desmethyl-imatinib related to induction of CYP3A4 and P-glycoprotein by St John’s wort extract [42].

#### Docetaxel

Docetaxel, a taxane antineoplastic agent, has also been reported to interact with St John’s wort [44]. Docetaxel is extensively metabolised and inactivated by CYP3A4 [44]. Goey et al. investigated the pharmacokinetic data of co-administering St John’s wort and docetaxel in cancer patients [45]. The open-label crossover study reported intravenously administering 135 mg docetaxel to ten cancer patients as part of a 21-day cycle [45]. St John’s wort at a dose of 300 mg, standardised to 0.36–0.84 mg hypericin and 9–19 mg hyperforin, was orally administrated three times daily between two cycles at day 7–21 [45]. The mean AUC_0–∞_ of docetaxel was significantly lower after pre-treatment with St John’s wort extract, whilst docetaxel clearance was significantly increased [45]. Clinically, the overall incidence of docetaxel-related adverse effects was lowered with concurrent use of St John’s wort [45]. The interaction was attributed to CYP3A4 induction by hyperforin in St John’s wort, resulting in increased docetaxel metabolism.

### Grapefruit juice

Grapefruit juice contains phytochemicals that can influence CYP3A-mediated metabolism, with clinical evidence of interactions with a range of medications [46]. The bioactive compound of grapefruit juice, naringin, has been identified as the likely perpetrator of the interactions via a mechanism-based inhibition of CYP3A4 [46]. Anti-cancer agents such as kinase inhibitors, sirolimus and etoposide are known to interact with grapefruit juice [47–50]. The inhibition of CYP3A enzymes may substantially reduce the first pass metabolism of drugs, particularly those with high hepatic extraction ratio, thereby increasing systemic exposure and potential toxicity of the drugs.

#### Tyrosine kinase inhibitors (nilotinib, sunitinib and imatinib)

Nilotinib, sunitinib and imatinib are tyrosine kinase inhibitors metabolised and inactivated by CYP3A4. In an open-label randomised crossover study with healthy participants (*n* = 21), the concurrent intake of 400 mg nilotinib with 240 mL double-strength grapefruit juice daily was associated with a higher nilotinib C_max_ by 60% and AUC by 29% compared to treatment with nilotinib alone [48].

Kimura et al. 2010 conducted a prospective study evaluating the potential interaction between grapefruit juice and imatinib in four patients with chronic myeloid leukaemia [47]. The intake of 400 mg imatinib and 250 mL grapefruit juice daily was not associated with any statistically significant changes in pharmacokinetics of imatinib [47]. A proposed explanation was due to a lower dose grapefruit juice used which was insufficient to inhibit hepatic CYP3A4 [47]. In a separate study, a lower dose of grapefruit juice was shown to preferentially inhibit intestinal CYP3A4, whilst a higher dose was shown to inhibit both intestinal and hepatic CYP3A4 [51]. Since the bioavailability of imatinib is almost 100%, inhibition of intestinal CYP3A4 would not significantly affect the absorption of imatinib [43]. It is unclear if high-dose grapefruit juice would affect imatinib pharmacokinetics.

#### Sirolimus

Sirolimus, also known rapamycin, is a mammalian target of rapamycin (mTOR) inhibitor, which has antiproliferative and immunosuppressive properties [52]. Sirolimus is used as an immunosuppressant for prophylaxis of rejection after renal transplant [52]. Sirolimus has an estimated bioavailability of 15%, and is extensively metabolised by intestinal and hepatic CYP3A4 enzyme [52]. Cohen EEW et al. analysed the sirolimus pharmacokinetic profile in cancer patients taking sirolimus alone (*n* = 40) and in a group (*n* = 41) who were given sirolimus and grapefruit juice [49]. Participants in sirolimus alone group took 1 mg/mL sirolimus oral solution once weekly in a dose escalation manner to achieve sirolimus blood AUC of 3810 ng h/mL, equivalent to 25 mg temsirolimus [49]. In the combination group, in which the same sirolimus regimen was used, grapefruit juice was initiated in week 2 at a dose of 240 mL once daily [49]. The minimum duration of trial is 8 weeks [49]. The study revealed that grapefruit juice significantly increased blood concentration of sirolimus by 350% [49]. Alone, an oral dose of 90 mg sirolimus was needed to achieve a target AUC of 3,810 ng.h/mL, whilst 25 mg sirolimus was required in the combination group [49].

#### Etoposide

Etoposide, a topoisomerase II inhibitor, is metabolised by CYP3A4 via *O*-demethylation [53]. Etoposide is often administered intravenously, but the oral bioavailability is estimated to be 50%, varying between individuals from 25 to 75% [54]. A study investigated the effect of co-administration of grapefruit juice on etoposide bioavailability in six patients with small cell lung cancer [50]. Participants were randomised to be sequentially treated with either 1-h intravenous 50 mg etoposide, 50 mg oral etoposide or 50 mg oral etoposide with 100 mL grapefruit juice pre-treatment [50]. The pre-treatment of grapefruit juice unexpectedly reduced etoposide bioavailability by 26.2% [50]. The underlying mechanism of interaction was not clear, but may partly be explained by inhibitory activity of phytochemical(s) in grapefruit juice (most likely two major flavonoids, naringin and hesperidin) [19] on the OATP2B1 transporter in the apical (brush border) membrane of enterocytes. Etoposide has been reported to be a substrate of both OATP1B1 and OATP2B1 [55], and hence inhibition of intestinal OATP2B1 due to co-administration with grapefruit juice may decrease the absorption of etoposide through enterocytes and lower its systemic exposure.

### Schisandra sphenanthera

*Schisandra sphenanthera* is a popular traditional Chinese medicine used for the management of hepatitis, Alzheimer’s disease, renal transplantation, osteoporosis and insomnia [56]. Meanwhile, other closely related species, *Schisandra chinensis* can be found in many Western countries [57]. The ethanolic extract of *Schisandra sphenanthera* is commercially available and known as Wuzhi tablet. Compounds (lignans) isolated from *Schisandra sphenanthera* have demonstrated anti-inflammatory, immunomodulatory and anti-cancer properties [56]. Previous in vitro studies highlighted *Schisandra* lignans as substrates, inhibitors and inducers of CYP3A enzymes [57], although the predominant net clinical outcome following a chronic exposure to *Schisandra sphenanthera* extract appears to be CYP3A inhibition.

#### Sirolimus

Li et al. investigated the combination of *Schisandra sphenanthera* extract and sirolimus in 18 healthy individuals [58]. Participants were administered with 2 mg sirolimus (or rapamycin), before and after a 13-day pre-treatment with *Schisandra sphenanthera* extract at a daily dose equivalent to 67.5 mg deoxyschizandrin [58]. The results showed a statistically significant increase in sirolimus AUC_0–∞_, C_max_ and t_max_ by 106.8%, 96.3% and 26.7%, respectively [58]. The apparent clearance of sirolimus was reduced by 38% when co-administered with *Schisandra sphenanthera* extract [58]. This suggests that dose adjustment of sirolimus may need to be considered if co-administration with *Schisandra sphenanthera* extract is desirable.

#### Tacrolimus

Similar to sirolimus, tacrolimus is an immunosuppressant that acts by inhibition of calcineurin and thereby inhibits T lymphocyte activation [59]. Tacrolimus is indicated for prevention of organ transplant rejection including kidney, heart, lung and pancreas [59]. As a drug with a narrow therapeutic index and a substrate of CYP3A, compounds that interact with tacrolimus could pose important clinical implications [59]. There is clinical evidence demonstrating the potential of *Schisandra sphenanthera* extract in increasing tacrolimus plasma concentrations [60–62].

Teng et al*.* explored the potential interaction between *Schisandra sphenanthera* capsules standardised to 11.3 mg of deoxyschizandrin and tacrolimus in 40 healthy volunteers [60]. Participants were administered with 2 mg tacrolimus followed by either 0, 1, 2, 6 or 8 capsules of *Schisandra sphenanthera* [60]. The results showed a dose-dependent increase in plasma tacrolimus associated with increasing *Schisandra sphenanthera* dose [60]. Relative to the control group, C_max_ of tacrolimus was significantly increased by 117%, 167%, 135% and 128% in 1, 2, 6 and 8 capsule of *Schisandra sphenanthera*, respectively, with a corresponding decrease in clearance [60]. The t_max_ was also found to be delayed by 71% and 108% at doses of 6 and 8 capsules of *Schisandra sphenanthera*, respectively [60]*.* Xin et al. conducted a similar study evaluating the interaction between tacrolimus and *Schisandra sphenanthera* [61]*.* Twelve healthy volunteers were administered with single dose of 2 mg tacrolimus before and after 13 days of *Schisandra sphenanthera* exposure, at a dose of three capsules (standardised with 11.3 mg deoxyschizandrin per capsule) twice daily [61]. Co-administration of *Schisandra sphenanthera* significantly increased tacrolimus AUC, C_max_ and t_max_ by 164%, 227.1% and 36.8%, respectively [61]. Another retrospective study analysed safety and efficacy of co-administration of tacrolimus and *Schisandra sphenanthera* in 194 Chinese renal transplant recipients [62]. The trough concentration over dosage was found to be significantly higher in Wuzhi group (*n* = 77) at timepoint 0, 1, 3, 9 and 12 months [62] relative to a control group (*n* = 177). No significant difference in liver and renal function tests was identified between groups [62].

Overall, these results indicate that *Schisandra sphenanthera* can significantly impact plasma concentration of tacrolimus and sirolimus. The clinical application of this interaction has been proposed as an opportunity for tacrolimus dose sparing and therefore a reduction in associated costs [63]. However, the extrapolation of this interaction should be interpreted with caution.

### Curcuma longa (turmeric)

#### Curcumin in combination with piperine

Curcumin is a polyphenol constituent extracted from the rhizome of *Curcuma longa*, also known as turmeric [64]. Curcumin possesses anti-inflammatory, antioxidant and immunomodulatory properties [64]. Its anti-cancer activity has also been demonstrated in a range of cancers including breast, lung, haematological, gastric, colorectal, pancreatic and hepatic cancer [64]. Due to low solubility, poor intestinal permeability and extensive first pass metabolism, curcumin has a low bioavailability [65]. Compounds such as piperine are often added to curcumin formulations to enhance bioavailability [65]. Curcumin has been shown to inhibit drug-metabolising enzymes, such as CYP3A4 [65].

##### Tamoxifen

Tamoxifen is a selective oestrogen receptor modulator used for the treatment and prevention of breast cancer recurrence [66]. Tamoxifen is metabolised sequentially by a combination CYP2D6 and CYP3A4 into the active metabolite endoxifen [66]. Endoxifen is inactivated via phase II metabolism by UGT and SULT [66]. The concurrent use of tamoxifen and curcumin with or without piperine was evaluated by Hussaarts et al. [67]. The randomised cross-over study recruited 16 patients with breast cancer who were taking 20–30 mg tamoxifen daily [67]. The study evaluated both tamoxifen and endoxifen pharmacokinetics in patients taking tamoxifen alone; tamoxifen with curcumin 1200 mg three times daily; or tamoxifen with both curcumin 1200 mg and 10 mg piperine three times daily [67]. Relative to tamoxifen monotherapy, patients treated with tamoxifen and curcumin had a lower tamoxifen AUC_0–24 h_ and trough concentration (C_trough_) by 8% and 7.1%, respectively [67]. The corresponding AUC_0–24 h_ and C_trough_ of endoxifen was reduced by 7.7 and 5.6%, respectively [67]. Patients who had a combination treatment of tamoxifen, curcumin and piperine, displayed further reduction of tamoxifen AUC_0–24 h_ and C_trough_ by 12.8% and 12.2%, respectively [67]. The endoxifen AUC_0–24 h_ and C_trough_ was also decreased by 12.4% and 12.4%, respectively [67]. In this study, extensive CYP2D6 metabolisers were observed to be more susceptible to the drug interaction [67].

#### Hydrastis canadensis (goldenseal)

Goldenseal (*Hydrastis canadensis*) is a herbaceous plant, native to North America [68]. It is traditionally used for the management of a variety of conditions including cancer [68].

##### CYP3A substrate

Although clinical studies reporting an interaction between anti-cancer agent and goldenseal are not available, there is evidence that goldenseal inhibits CYP3A enzyme activity [69, 70]. The CYP enzyme inhibitory activity of 2700 mg daily goldenseal root extract was evaluated by Gurley et al. in 12 healthy volunteers [69]. Midazolam and debrisoquine were used as probe drugs to evaluate the activity (phenotype) of CYP3A and CYP2D6 enzyme, respectively [69]. The results showed a 28-day supplementation of goldenseal had reduced CYP3A and CYP2D6 activity by 40% [69]. Similar results were demonstrated in an open-label randomised clinical study in 16 healthy participants [70]. Concomitant administration of goldenseal root extract at a dose of 1323 mg three times daily for 14 days led to a significant alteration in midazolam pharmacokinetic parameters, including increased AUC_0–∞_, half-life, C_max_ and reduced apparent clearance, consistent with inhibition of intestinal and hepatic CYP3A enzymes [70].

Despite the lack of direct clinical evidence evaluating interaction between goldenseal and anti-cancer agents, the current data may be extrapolated to other CYP3A substrate anti-cancer agents including kinase inhibitors. Until further evidence becomes available, co-administration of goldenseal and CYP3A substrate should be avoided.

## Clinically significant pharmacodynamic interactions

The anti-cancer properties of a range of NPs and phytochemicals have been evaluated in in vitro and xenograft models. However, strong clinical evidence to support the use of NPs for the management of cancer is lacking. In line with that, clinical data addressing the pharmacodynamic interactions of NPs and anti-cancer agents are scarce. Hence, this review will focus on the pharmacodynamic-based interactions that modulate the adverse effect of anti-cancer drugs, such as chemotherapy-induced diarrhoea.

### Hangeshashinto (TJ-14)

Hangeshashinto, also known as TJ-14, is a traditional Japanese medicine composed of extracts from seven herbs including *Pinellia* tuber, *Scutellariae* radix, *Glycyrrhizae* radix, *Ziziphi* fructus, *Ginseng* radix, *Zingiberis processum* rhizoma and *Coptidis* rhizome [71]. It is used for the management of gastrointestinal conditions such as gastroenteritis [71]. Several clinical studies have evaluated the effects of TJ-14 in alleviating anti-cancer agent-induced oral mucositis, enteritis and diarrhoea [71–76].

#### Afatinib

Ichiki et al. assessed the prophylactic effect of TJ-14 on afatinib-induced diarrhoea and oral mucositis in 29 non-small cell lung cancer patients [72]. Participants were administered 7.5 g daily TJ-14 (gargled and swallowed) and 40 mg afatinib daily for 4 weeks [72]. Incidence of afatinib-associated all-grade diarrhoea was 68.9% and ≥ grade 3 diarrhoea was 3.4%. This incidence was lower than that previously reported in phase III clinical trials of afatinib monotherapy, where the all-grade diarrhoea was 88.3 to 95%, whilst ≥ grade 3 diarrhoea was 5.4–14.4% [77, 78]. The authors suggested that this encouraging result may support the potential use of TJ-14 as a prophylactic treatment for afatinib-induced diarrhoea [72].

#### Irinotecan

A randomised comparative trial investigated the potential beneficial effect of TJ-14 in the management of irinotecan-induced diarrhoea [73]. Patients with advanced non-small cell lung cancer receiving irinotecan and cisplatin (*n* = 41) were administered TJ-14 7.5 g daily 3 days prior to chemotherapy and continued for 21 days [73]. The TJ-14 group had showed a significant lowered diarrhoea grades and incidence of grade 3 and 4 diarrhoea, relative to a control group [73]. No difference was identified in the frequency and duration of diarrhoea between control and TJ-14 group [73].

##### FOLFOX, FOLFIRI and XELOX chemotherapy regimen

The same dose of 7.5 mg daily TJ-14 was evaluated in a double-bind randomised phase II trial in 93 colorectal cancer patients using either of the three regimen: (1) folinic acid, fluorouracil and oxaliplatin (FOLFOX), (2) folinic acid, fluorouracil and irinotecan (FOLFIRI), or (3) capecitabine and oxaliplatin (XELOX) [74]. Participants were instructed to use TJ-14 on the first day of chemotherapy and continued for 14 days [74]. TJ-14 treatment was found to lower the risk of ≥ grade 2 oral mucositis incidence by 15% and was associated with a statistically significant reduction of ≥ 2 oral mucositis duration [74].

#### Fluoropyrimidine-based chemotherapy for gastric cancer

The efficacy of TJ-14 in chemotherapy-induced mucositis in gastric cancer patient population was evaluated by Aoyama et al. [75]. Participants (*n* = 91) in this randomised double-blind placebo-controlled phase II trial received chemotherapy including tegafur, 5-chloro-2-4-dihydroxypyridine and oxonic acid (S-1) monotherapy or S-1 plus cisplatin or S-1 plus paclitaxel or paclitaxel monotherapy or S-1 plus docetaxel or docetaxel monotherapy or irinotecan plus cisplatin or irinotecan monotherapy [75]. TJ-14 7.5 g daily was administered from the first day to final day of treatment for a total period of 2–6 weeks, depending on the chemotherapy protocol [75]. No statistically significant difference was found in incidence of ≥ grade 2 oral mucositis between the placebo and the TJ-14 treatment group [75]. The researchers concluded that the lack of efficacy of TJ-14 was likely due to dose reduction of chemotherapy between cycles [75]. However, a trend was observed in oral mucositis risk reduction by TJ-14 amongst patients who developed grade 1 oral mucositis during the screening cycle [75].

#### Fluoropyrimidine-based chemotherapy for gastric and colorectal cancer

Nishikawa et al. evaluated the efficacy of TJ-14 in the prevention and/or treatment of chemotherapy-induced oral mucositis in gastric and colorectal cancer based on the data from phase II randomised clinical trials by Matsuda et al. and Aoyama et al. [74–76]. Analysis of the pooled data of 181 patients receiving 7.5 g daily TJ-14 with chemotherapy for 4–6 weeks did not lead to a statistically significant difference in the incidence of ≥ grade 2 oral mucositis between treatment and placebo group [76]. However, treatment with TJ-14 was associated with a significant reduction in duration of severe ≥ grade 2 oral mucositis [76]. The median time to remission of ≥ grade 2 to < grade 1 oral mucositis was 8 days in TJ-14 treatment group, compared to 15 days in the placebo group [76].

In summary, TJ-14 has a potential beneficial interaction with anti-cancer agent in the management of chemotherapy-induced gastrointestinal disturbance. However, more extensive clinical data are required to support the use of TJ-14 as adjunct therapy to help manage the gastrointestinal adverse effects of anti-cancer drugs.

### *Panax ginseng*, *Panax quinquefolium* and *Eleutherococcus senticosus*, (ginseng)

Ginseng is a herb belonging to the Araliaceae family [79]. The main ginseng species used medicinally are *Panax ginseng* also known as Korean ginseng, *Panax quinquefolium* also known as American ginseng, and *Eleutherococcus senticosus*, also known as Siberian ginseng [79]. Korean and American ginseng are commonly used for the management of cancer-related fatigue. Anti-cancer treatments are a common cause of cancer-related fatigue [80]. The co-administration of ginseng and anti-cancer agent may pose a potential pharmacodynamic interaction.

#### *Panax quinquefolius* (American ginseng)

A double-blind randomised controlled trial (*n* = 364) evaluated the efficacy and toxicity of 2,000 mg American ginseng daily for cancer-related fatigue [81]. Using Multidimensional Fatigue Symptom Inventory-Short Form (MFSI-SF), a statistically significant improvement in the fatigue score of the ginseng group (*n* = 138, score change = 20) compared to placebo group (*n* = 133, scores change = 10.3) after 8 weeks was observed [81]. In a stratified analysis, participants who were concurrently receiving both ginseng and conventional cancer treatment (conventional cancer treatment *n* = 83; tamoxifen *n* = 23; aromatase inhibitor *n* = 27; antiandrogen *n* = 2) demonstrated a statistically significant improvement in fatigue score at both week 4 and 8 compared to placebo group (*n* = 83) [81].

#### *Panax ginseng* (Korean ginseng)

Jiang et al. investigated the concomitant use of fermented Korean red ginseng extract in non-small cell lung cancer patients [82]. Thirty-four patients had received gemcitabine with cisplatin and fermented Korean red ginseng at 3000 mg daily, whilst twenty-six patients received only the chemotherapy drugs for 60 days [82]. The study highlighted a significant positive improvement in fatigue scores, cancer-related symptoms, psychological status, physical conditions, quality of life and chemotherapy-induced adverse effect in treatment group compared to those receiving chemotherapy alone [82]. However, no significant changes in tumour markers including carcinoembryonic antigen, neuron-specific enolase and cytokeratin-19 fragments were identified between treatment and chemotherapy alone group.

To date, there are unclear and insufficient clinical data to support the safety and efficacy of both American and Korean ginseng for the management of cancer-related fatigue. Due to limited clinical studies and the heterogeneity of the used anti-cancer agents, cancer types and ginseng formulation, it was not straightforward to clearly identify pharmacodynamic-based interactions between ginseng and anti-cancer drugs.

### PHY906

PHY906 is a natural product comprising *Scutellaria baicalensis*, *Glycyrrhiza uralensis*, *Paeonia lactiflora*, and *Ziziphus jujuba*, a combination of which has been known in the traditional Chinese medicine for their putative effects in alleviating nausea and diarrhoea [83]. The anti-diarrhoeal effect of PHY906 was evaluated in 17 patients with advanced colorectal cancer receiving treatment with irinotecan (125 mg/m^2^), leucovorin (20 mg/m^2^) and fluorouracil (500 mg/m^2^) weekly on a 4-week on and 2-week off regimen over a 6-week cycle. Concurrent administration with PHY906 (1.2–2.4 g of the extract daily) for a median duration of 16 days appeared to reduce the overall incidence of grade 3 or 4 diarrhoea and the corresponding use of loperamide, with no effect on pharmacokinetics [84]. This potential beneficial effect in ameliorating the chemotherapy-induced diarrhoea was assumed to be due to the attenuation of intestinal inflammation and stimulation of recovery of the damaged mucosal intestinal cells [83]. PHY906 given at an 800-mg twice daily dosing regimen also seemed to improve the safety profile of capecitabine, an oral prodrug of fluorouracil, in patients with hepatocellular carcinoma (HCC) [85]. However, no direct comparison with the chemotherapy regimen alone was possible due to the lack of placebo control group in this study. Interestingly, an orphan drug designation was granted to PHY906 by the US Food and Drug Administration (FDA) in 2018 for the therapeutic indication of HCC. This has led to a number of ongoing clinical trials that further explore the inclusion of PHY906 in HCC therapy regimens, e.g. in combination with sorafenib or capecitabine, with the purported aim to help manage the chemotherapy-induced gastrointestinal toxicity [85].

### Wheat grass juice

Wheat grass juice is derived from the mature sprouts of wheat seeds (*Triticum aestivum*), with putative antioxidant properties related to a high content of flavonoids and phenolic compounds [86]. A placebo-controlled pilot study evaluated the combination of wheat grass juice with the FAC regimen (fluorouracil 500 mg/m^2^, adriamycin or doxorubicin 50 mg/m^2^, and cyclophosphamide 500 mg/m^2^ administered once every 3 weeks) in women with breast cancer (30 patients each for the control and combination treatment group) [87]. Daily ingestion of wheat grass juice (60 mL per day) during the first three cycles of the chemotherapy regimen significantly reduced the incidence of grade 3 or 4 leukopaenia compared to the control arm (17 vs. 43%), whilst no significant difference in the incidence of neutropaenia was observed. Interestingly, the need for supportive therapy with granulocyte colony stimulating factor (G-CSF) was also substantially lower in patients receiving the juice compared to the control FAC group [87]. The response rate to the anti-cancer regimen and time-to-tumour progression were also evaluated with and without co-administration of wheat grass juice, with a median follow-up duration of 23 months. These responses were reported as not being significantly affected by the juice. Despite the encouraging ameliorative effect of wheat grass juice on treatment-related haematological toxicity, further clinical evaluation with a larger number of cancer patients is desirable (Table 1).

**Table 1:** Clinically significant interactions—pharmacokinetics

Natural product (dosage)	Anti-cancer agent (dose)	Proposed mechanism of interaction	Study population	Outcome	Reference
St John’s wort (*Hypercium perforatum*) (300 mg three times daily)	Irinotecan (350 mg/m^2^)	Induction of CYP3A4	Colorectal cancer (*n* = 2)	Reduced formation of the active compound SN-38	Mathijssen et al. 2002 [38]
			lung cancer (*n* = 2)		
			sarcoma (*n* = 1)		
	Imatinib (400 mg)	Induction of CYP3A4 and P-glycoprotein	Healthy individuals (*n* = 10)	Reduced absorption and increased elimination of imatinib, increased serum imatinib metabolite *N*-desmethyl-imatinib	Smith et al. 2004 [41]
	Docetaxel (135 mg intravenously)	Induction of CYP3A4	Bladder cancer (*n* = 6)	Reduced docetaxel plasma level and increased clearance	Goey et al. 2014 [45]
			Lung cancer (*n* = 3)		
			Ovarian cancer (*n* = 1)		
			Ureteral cancer (*n* = 1)		
Grapefruit juice (100–600 mL daily)^^^	Nilotinib (400 mg daily)	Inhibition of hepatic CYP3A4	Healthy individuals (*n* = 21)	Increased serum nilotinib level	Yin et al. 2010 [48]
	Sirolimus (dose escalation manner to achieve sirolimus AUC of 3810 ng h/mL)	Inhibition of hepatic CYP3A4	Cancer patients* (*n* = 41)	Significantly increased whole blood concentrations of sirolimus	Cohen et al. 2012 [49]
	Etoposide (50 mg orally)	Unclear mechanism, but possibly inhibition of intestinal OATP2B1	Small cell lung cancer (*n* = 6)	Decreased bioavailability of etoposide	Reif et al. 2002 [50]
*Schisandra sphenanthera* (three capsules of *Schisandra sphenanthera* extract containing 11.25 mg deoxyschizandrin twice daily for 13 days)	Sirolimus (2 mg single dose)	CYP3A4 inhibition	Healthy individuals (*n* = 18)	Enhanced bioavailability and reduced elimination	Li et al. 2012 [58]
Curcumin in combination with piperine (curcumin 1200 mg three times daily, piperine 10 mg three times daily)	Tamoxifen (20–30 mg daily)	Unknown	Breast (*n* = 16)	Decreased tamoxifen and endoxifen AUC_0–24 h_ and C_trough_	Hussaarts et al. 2019 [67]

*Colorectal sarcoma, pancreas, ovarian, head and neck, lung, thyroid, prostate and oesophagus cancer

^^^240 mL double-strength grapefruit juice once daily

Studies reporting anti-cancer drug–natural product interactions with no clinical relevance are summarised in Table 2.

**Table 2:** Studies reporting drug–NP interactions of no clinical importance

Herbal product; formulation (dosage)	Anti-cancer agent (dosage)	Study population characteristics (*n*) duration	Outcome	Reference
Grapefruit juice (200 mL 3 times daily)	Sunitinib (20–25 mg daily)	Renal cancer or gastrointestinal stromal tumour (*n* = 8)	Increased bioavailability of sunitinib, non-clinically relevant	Van Erp et al. 2011 [88]
Curcumin phosphatidylcholine complex (1–4 g daily)	Irinotecan (200 mg/m^2^on day 1 and 15 of 28 day-cycle)	Advanced solid tumours	No significant changes in plasma exposure and other pharmacokinetic properties of irinotecan and its metabolites	Gbolahan OB et al. 2022 [89]
Green tea; capsule containing 150 mg EGCG equivalent to 5–6 cups of regular green tea (1000 mg twice daily)	Tamoxifen (20 or 40 mg daily)	Women with breast cancer (*n* = 14); 14 days	Tamoxifen and endoxifen pharmacokinetics were not affected by green tea supplements	Braal et al. 2020 [90]
Garlic; tablet containing 3600 µg (600 mg twice daily)	Docetaxel (30 mg/m^2^ weekly)	Women with breast cancer (*n* = 10); 12 days between day 5 to 17	Garlic supplementation did not significantly affect the disposition of the CYP3A4 substrate drug docetaxel. However, it could not be excluded that garlic decreases the clearance of docetaxel in patients with CYP3A5*1/*1 allele	Cox et al. 2006 [34]
Medicinal cannabis; 1 g/L herbal tea containing 18% THC and 0.8% CBD (once daily)	Irinotecan (90 min IV infusion 600 mg) or docetaxel (1-h IV infusion, at a fixed dose of 180 mg)	Cancer patients being treated with irinotecan (*n* = 12) or docetaxel (*n* = 12); 15 days	Co-administration of medicinal cannabis herbal tea in cancer patients treated with irinotecan or docetaxel did not significantly influence plasma pharmacokinetics of these drugs	Engels et al. 2007 [91]
*Echinacea purpurea*; extract drops containing 95% aerial parts and 5% roots (20 drops three times daily)	Docetaxel (135 mg IV infusion every 3 weeks)	Cancer patients (*n* = 10); 14 days between docetaxel cycle	At the recommended dose and schedule of a commercially available *E. purpurea* extract had no statistically significant interference with docetaxel pharmacokinetics	Goey et al. 2013 [92]
Soy food and soy products	Tamoxifen	Patients with breast cancer	No evidence supporting the use of soy food products would detrimentally affect pharmacological activity of tamoxifen	Guha et al. 2009 [93]
European mistletoe (*Viscum album*) extract (20–250 mg daily by subcutaneous injection)	Gemcitabine (750 mg/m^2^ intravenously) on day 1 and 8 of a 3-week cycle	Patients with advanced solid tumours (*n* = 20)	Systemic exposure to gemcitabine was not significantly affected by co-administration with mistletoe extract	Mansky et al. 2013 [94]
Chrysin; (250 mg twice daily)	Irinotecan (IV 350 mg/m^2^ every 3 weeks)	Patients with metastatic colorectal cancer (*n* = 20); 1 week	Combining chrysin with irinotecan may be a safe and potentially useful means of preventing diarrhoea	Tobin et al. 2005 [95]
Fucoidan (sulphated carbohydrates derived from marine brown algae, *Undaria pinnatifida*; 500 mg twice daily for a 3-week period)	Letrozole (2.5 mg daily) and tamoxifen (20 mg daily) for at least 4 weeks	Women with breast cancer being treated with either letrozole or tamoxifen (*n* = 10 each)	Plasma trough concentrations of letrozole, tamoxifen and its metabolite, endoxifen appeared to be unaffected by the concurrent use of fucoidan. The combination was well tolerated with no adverse effect related to the use of the natural product was reported	Tocaciu et al. 2018 [96]
Milk thistle; seed extract containing 80% silymarin (three times daily)	Irinotecan (125 mg/m^2^ IV infusion once a week)	Cancer patients (*n* = 6); milk thistle was taken for 4 days before the second irinotecan dose and continued for 14 days	Milk thistle possessed little risk of interfering with pharmacokinetics of chemotherapeutic agents that are substrates of CYP3A4 and UGT1A1	Van Erp et al. 2005 [97]
*Ginkgo biloba*; EGb 761 (120 mg twice daily)	Tamoxifen; anastrozole; letrozole	Women living with early stage breast cancer tamoxifen(*n* = 20) anastrozole (*n* = 20) letrozole (*n* = 20); 3 weeks	Co-administration of EGb761 was unlikely to lead to clinically significant interaction for women who are receiving hormonal treatment for breast cancer	Vardy et al. 2013 [98]
Green tea extract (contained 60% EGCG; 500 mg twice daily for 7 days)	Nintedanib (100–150 mg per oral twice daily)	Patients with pulmonary fibrosis (*n* = 28)	Co-administration of green tea extract led to a modest yet significant reduction (with a median of − 21%) in systemic exposure to nintedanib. However, this might be of little clinical importance given that the range of therapeutic concentration of the drug is likely to be wide	Veerman et al. 2022 [99]

*CBD* cannabidiol, *EGCG* epigallocatechin-3-gallate, *IV* intravenous, *THC* delta-9-tetrahydrocannabinol

## Conclusion

This review provides an overview of pharmacokinetic and pharmacodynamic interaction mechanisms and clinically important interactions between NPs and anti-cancer drugs. To date, clinical studies investigating pharmacokinetic interactions provide evidence that negative treatment outcomes may occur when *Hypericum perforatum*, grapefruit juice, *Schisandra sphenanthera, Curcuma longa,* or *Hydrastis canadensis* are taken concurrently with common cancer drugs. Conversely, pharmacodynamic interactions between Hangeshashinto (TJ-14) and some cancer drugs have been found to reduce the side effects of diarrhoea and oral mucositis. Overall research in this area is limited. Further clinical studies investigating potential interactions involving NP commonly used by people living with cancer are critical to understanding the scope and relevance of any benefits or harms within a clinical setting.

## Supplementary Information

Below is the link to the electronic supplementary material.

Supplementary file1 (DOCX 17 KB)

Supplementary file2 (PDF 2134 KB)

## Data Availability

The data reported in this review article can be accessed in the respective references cited.

## References

[1] National Center for Complementary and Integrative Health (2022) Complementary, alternative, or integrative health: What’s in a name? National Center for Complementary and Integrative Health. https://www.nccih.nih.gov/health/complementary-alternative-or-integrative-health-whats-in-a-name. Accessed 14 Sep 2022

[2] Kappauf H, Leykauf-Ammon D, Bruntsch U, Horneber M, Kaiser G, Büschel G, Gallmeier WM (2000). Use of and attitudes held towards unconventional medicine by patients in a department of internal medicine/oncology and haematology. Support Care Cancer.

[3] Oh B, Butow P, Mullan B, Beale P, Pavlakis N, Rosenthal D, Clarke S (2010). The use and perceived benefits resulting from the use of complementary and alternative medicine by cancer patients in Australia. Asia Pac J Clin Oncol.

[4] Hlubocky FJ, Ratain MJ, Wen M, Daugherty CK (2007). Complementary and alternative medicine among advanced cancer patients enrolled on phase I trials: a study of prognosis, quality of life, and preferences for decision making. J Clin Oncol.

[5] Kumar NB, Hopkins K, Allen K, Riccardi D, Besterman-Dahan K, Moyers SJCC (2002). Use of complementary/integrative nutritional therapies during cancer treatment: implications in clinical practice. Cancer Control.

[6] Chan WJJ, McLachlan AJ, Wheate NJ, Harnett JE (2018). An evaluation of garlic products available in Australian pharmacies–from the label to the laboratory. J Herb Med.

[7] Cohen M, Hunter J (2017). Complementary medicine products: interpreting the evidence base. Intern Med J.

[8] Barnes J (2003). Quality, efficacy and safety of complementary medicines: fashions, facts and the future. Part I. Regulation and quality. Br J Clin Pharmacol.

[9] Carmona F, Pereira AMS (2013). Herbal medicines: old and new concepts, truths and misunderstandings. Rev Bras Farmacogn.

[10] Coxeter PD, McLachlan AJ, Duke CC, Roufogalis BD (2004). Herb-drug interactions: an evidence based approach. Curr Med Chem.

[11] Niu J, Straubinger RM, Mager DE (2019). Pharmacodynamic drug-drug interactions. Clin Pharmacol Ther.

[12] Florence A, Attwood D (1998). Physicochemical drug interactions and incompatibilities. Physicochemical principles of pharmacy.

[13] Ekhart C, Rodenhuis S, Smits PH, Beijnen JH, Huitema AD (2009). An overview of the relations between polymorphisms in drug metabolising enzymes and drug transporters and survival after cancer drug treatment. Cancer Treat Rev.

[14] Hartmann JT, Haap M, Kopp HG, Lipp HP (2009). Tyrosine kinase inhibitors—a review on pharmacology, metabolism and side effects. Curr Drug Metab.

[15] Mizuno N, Niwa T, Yotsumoto Y, Sugiyama Y (2003). Impact of drug transporter studies on drug discovery and development. Pharmacol Rev.

[16] Lin JH, Yamazaki MJCp (2003). Role of P-glycoprotein in pharmacokinetics. Clin Pharmacokinet.

[17] Saneja A, Khare V, Alam N, Dubey RD, Gupta PN (2014). Advances in P-glycoprotein-based approaches for delivering anticancer drugs: pharmacokinetic perspective and clinical relevance. Expert Opin Drug Deliv.

[18] Stieger B, Mahdi ZM, Jäger W (2017). Intestinal and hepatocellular transporters: therapeutic effects and drug interactions of herbal supplements. Annu Rev Pharmacol Toxicol.

[19] Dolton MJ, Roufogalis BD, McLachlan AJ (2012). Fruit juices as perpetrators of drug interactions: the role of organic anion-transporting polypeptides. Clin Pharmacol Ther.

[20] Chrubasik-Hausmann S, Vlachojannis J, McLachlan AJ (2019). Understanding drug interactions with St John’s wort (*Hypericum perforatum* L.): impact of hyperforin content. J Pharm Pharmacol.

[21] Rodrigues AD, Lai Y, Shen H, Varma MVS, Rowland A, Oswald S (2020). Induction of human intestinal and hepatic organic anion transporting polypeptides: where is the evidence for its relevance in drug-drug interactions?. Drug Metab Dispos.

[22] Greenblatt DJ (2017). Mechanisms and consequences of drug-drug interactions. Clin Pharmacol Drug Dev.

[23] Nicolussi S, Drewe J, Butterweck V, Meyer Zu Schwabedissen HE (2020). Clinical relevance of St. John’s wort drug interactions revisited. Br J Pharmacol.

[24] Yang J, Kjellsson M, Rostami-Hodjegan A, Tucker GT (2003). The effects of dose staggering on metabolic drug-drug interactions. Eur J Pharm Sci.

[25] Paine MF, Widmer WW, Hart HL, Pusek SN, Beavers KL, Criss AB, Brown SS, Thomas BF, Watkins PB (2006). A furanocoumarin-free grapefruit juice establishes furanocoumarins as the mediators of the grapefruit juice-felodipine interaction. Am J Clin Nutr.

[26] Fontana E, Dansette PM, Poli SM (2005). Cytochrome p450 enzymes mechanism based inhibitors: common sub-structures and reactivity. Curr Drug Metab.

[27] Brantley SJ, Argikar AA, Lin YS, Nagar S, Paine MF (2014). Herb-drug interactions: challenges and opportunities for improved predictions. Drug Metab Dispos.

[28] Cox EJ, Tian DD, Clarke JD, Rettie AE, Unadkat JD, Thummel KE, McCune JS, Paine MF (2021). Modeling Pharmacokinetic natural product-drug interactions for decision-making: a NaPDI center recommended approach. Pharmacol Rev.

[29] Zhang H, Bu F, Li L, Jiao Z, Ma G, Cai W, Zhuang X, Lin HS, Shin JG, Xiang X (2018). Prediction of drug-drug interaction between tacrolimus and principal ingredients of Wuzhi Capsule in Chinese healthy volunteers using physiologically-based pharmacokinetic modelling. Basic Clin Pharmacol Toxicol.

[30] McDonald MG, Tian DD, Thummel KE, Paine MF, Rettie AE (2020). Modulation of major human liver microsomal cytochromes P450 by Component alkaloids of goldenseal: time-dependent inhibition and allosteric effects. Drug Metab Dispos.

[31] Malki MA, Pearson ER (2020). Drug-drug-gene interactions and adverse drug reactions. Pharmacogenom J.

[32] Imanaga J, Kotegawa T, Imai H, Tsutsumi K, Yoshizato T, Ohyama T, Shirasaka Y, Tamai I, Tateishi T, Ohashi K (2011). The effects of the SLCO2B1 c.1457C > T polymorphism and apple juice on the pharmacokinetics of fexofenadine and midazolam in humans. Pharmacogenet Genom.

[33] Mougey EB, Lang JE, Wen X, Lima JJ (2011). Effect of citrus juice and SLCO2B1 genotype on the pharmacokinetics of montelukast. J Clin Pharmacol.

[34] Cox MC, Low J, Lee J, Walshe J, Denduluri N, Berman A, Permenter MG, Petros WP, Price DK, Figg WD, Sparreboom A, Swain SM (2006). Influence of garlic (*Allium sativum*) on the pharmacokinetics of docetaxel. Clin Cancer Res.

[35] Pirl WF (2004). Evidence report on the occurrence, assessment, and treatment of depression in cancer patients. J Natl Cancer Inst Monogr.

[36] Redvers A, Laugharne R, Kanagaratnam G, Srinivasan GJPB (2001). How many patients self-medicate with St John’s wort?. Psychiatr Bull.

[37] Dürr D, Stieger B, Kullak-Ublick GA, Rentsch KM, Steinert HC, Meier PJ, Fattinger K (2000). St John's Wort induces intestinal P-glycoprotein/MDR1 and intestinal and hepatic CYP3A4. Clin Pharmacol Ther.

[38] Mathijssen RH, Verweij J, de Bruijn P, Loos WJ, Sparreboom A (2002). Effects of St. John’s wort on irinotecan metabolism. J Natl Cancer Inst.

[39] Mathijssen RH, van Alphen RJ, Verweij J, Loos WJ, Nooter K, Stoter G, Sparreboom A (2001). Clinical pharmacokinetics and metabolism of irinotecan (CPT-11). Clin Cancer Res.

[40] Parvez MM, Basit A, Jariwala PB, Gáborik Z, Kis E, Heyward S, Redinbo MR, Prasad B (2021). Quantitative investigation of irinotecan metabolism, transport, and gut microbiome activation. Drug Metab Dispos.

[41] Smith P, Bullock JM, Booker BM, Haas CE, Berenson CS, Jusko WJ (2004). The influence of St. John’s wort on the pharmacokinetics and protein binding of imatinib mesylate. Pharmacotherapy.

[42] Frye RF, Fitzgerald SM, Lagattuta TF, Hruska MW, Egorin MJ (2004). Effect of St John’s wort on imatinib mesylate pharmacokinetics. Clin Pharmacol Ther.

[43] Peng B, Lloyd P, Schran H (2005). Clinical pharmacokinetics of imatinib. Clin Pharmacokinet.

[44] Baker SD, Sparreboom A, Verweij JJCp (2006). Clinical pharmacokinetics of docetaxel. Clin Pharmacokinet.

[45] Goey AK, Meijerman I, Rosing H, Marchetti S, Mergui-Roelvink M, Keessen M, Burgers JA, Beijnen JH, Schellens JH (2014). The effect of St John’s wort on the pharmacokinetics of docetaxel. Clin Pharmacokinet.

[46] Bailey DG, Malcolm J, Arnold O, Spence D (1998). Grapefruit juice–drug interactions. Br J Clin Pharmacol.

[47] Kimura S, Kako S, Wada H, Sakamoto K, Ashizawa M, Sato M, Terasako K, Kikuchi M, Nakasone H, Okuda S, Yamazaki R, Oshima K, Nishida J, Watanabe T, Kanda Y (2011). Can grapefruit juice decrease the cost of imatinib for the treatment of chronic myelogenous leukemia?. Leuk Res.

[48] Yin OQ, Gallagher N, Li A, Zhou W, Harrell R, Schran H (2010). Effect of grapefruit juice on the pharmacokinetics of nilotinib in healthy participants. J Clin Pharmacol.

[49] Cohen EE, Wu K, Hartford C, Kocherginsky M, Eaton KN, Zha Y, Nallari A, Maitland ML, Fox-Kay K, Moshier K, House L, Ramirez J, Undevia SD, Fleming GF, Gajewski TF, Ratain MJ (2012). Phase I studies of sirolimus alone or in combination with pharmacokinetic modulators in advanced cancer patients. Clin Cancer Res.

[50] Reif S, Nicolson MC, Bisset D, Reid M, Kloft C, Jaehde U, McLeod HL (2002). Effect of grapefruit juice intake on etoposide bioavailability. Eur J Clin Pharmacol.

[51] Veronese ML, Gillen LP, Burke JP, Dorval EP, Hauck WW, Pequignot E, Waldman SA, Greenberg HE (2003). Exposure-dependent inhibition of intestinal and hepatic CYP3A4 in vivo by grapefruit juice. J Clin Pharmacol.

[52] Sehgal SN (2003). Sirolimus: its discovery, biological properties, and mechanism of action. Transplant Proc.

[53] Zhuo X, Zheng N, Felix CA, Blair IA (2004). Kinetics and regulation of cytochrome P450-mediated etoposide metabolism. Drug Metab Dispos.

[54] Slevin MLJC (1991). The clinical pharmacology of etoposide. Cancer.

[55] Fahrmayr C, König J, Auge D, Mieth M, Fromm MF (2012). Identification of drugs and drug metabolites as substrates of multidrug resistance protein 2 (MRP2) using triple-transfected MDCK-OATP1B1-UGT1A1-MRP2 cells. Br J Pharmacol.

[56] Huang S, Zhang D, Li Y, Fan H, Liu Y, Huang W, Deng C, Wang W, Song X (2021). *Schisandra sphenanthera*: a comprehensive review of its botany, phytochemistry, pharmacology, and clinical applications. Am J Chin Med.

[57] Jackson JP, Freeman KM, Friley WW, Herman AG, Black CB, Brouwer KR, Roe AL (2017). Prediction of clinically relevant herb-drug clearance interactions using sandwich-cultured human hepatocytes: *Schisandra* spp. Case Study Drug Metab Dispos.

[58] Li R, Guo W, Fu Z, Ding G, Wang Z, Fu H (2012). A study about drug combination therapy of *Schisandra sphenanthera* extract and Rapamycin in healthy subjects. Can J Physiol Pharmacol.

[59] Plosker GL, Foster RHJD (2000). Tacrolimus: a further update of its pharmacology and therapeutic use in the management of organ transplantation. Tacrolimus.

[60] Teng F, Wang W, Zhang W, Qu J, Liu B, Chen J, Liu S, Li M, Chen W, Wei H (2022). Effect of hepar-protecting Wuzhi capsule on pharmacokinetics and dose-effect character of tacrolimus in healthy volunteers. Biopharm Drug Dispos.

[61] Xin HW, Wu XC, Li Q, Yu AR, Zhu M, Shen Y, Su D, Xiong L (2007). Effects of *Schisandra sphenanthera* extract on the pharmacokinetics of tacrolimus in healthy volunteers. Br J Clin Pharmacol.

[62] Cheng F, Li Q, Wang J, Zeng F, Zhang Y (2021). Effects and safety evaluation of Wuzhi capsules combined with tacrolimus for the treatment of kidney transplantation recipients. J Clin Pharm Ther.

[63] Li J, Chen S, Qin X, Fu Q, Bi H, Zhang Y, Wang X, Liu L, Wang C, Huang M (2017). Wuzhi tablet (*Schisandra sphenanthera* extract) is a promising tacrolimus-sparing agent for renal transplant recipients who are CYP3A5 expressers: a two-phase prospective study. Drug Metab Dispos.

[64] Giordano A, Tommonaro G (2019). Curcumin and cancer. Nutrients.

[65] Adiwidjaja J, McLachlan AJ, Boddy AV (2017). Curcumin as a clinically-promising anti-cancer agent: pharmacokinetics and drug interactions. Expert Opin Drug Metab Toxicol.

[66] Klein DJ, Thorn CF, Desta Z, Flockhart DA, Altman RB, Klein TE (2013). PharmGKB summary: tamoxifen pathway, pharmacokinetics. Pharmacogenet Genom.

[67] Hussaarts K, Hurkmans DP, Oomen-de Hoop E, van Harten LJ, Berghuis S, van Alphen RJ, Spierings LEA, van Rossum-Schornagel QC, Vastbinder MB, van Schaik RHN, van Gelder T, Jager A, van Leeuwen RWF, Mathijssen RHJ (2019). Impact of curcumin (with or without piperine) on the pharmacokinetics of tamoxifen. Cancers (Basel).

[68] Mandal SK, Maji AK, Mishra SK, Ishfaq PM, Devkota HP, Silva AS, Das N (2020). Goldenseal (*Hydrastis canadensis* L.) and its active constituents: a critical review of their efficacy and toxicological issues. Pharmacol Res.

[69] Gurley BJ, Gardner SF, Hubbard MA, Williams DK, Gentry WB, Khan IA, Shah A (2005). In vivo effects of goldenseal, kava kava, black cohosh, and valerian on human cytochrome P450 1A2, 2D6, 2E1, and 3A4/5 phenotypes. Clin Pharmacol Ther.

[70] Gurley BJ, Swain A, Hubbard MA, Hartsfield F, Thaden J, Williams DK, Gentry WB, Tong Y (2008). Supplementation with goldenseal (*Hydrastis canadensis*), but not kava kava (*Piper methysticum*), inhibits human CYP3A activity in vivo. Clin Pharmacol Ther.

[71] Yamashita T, Araki K, Tomifuji M, Kamide D, Tanaka Y, Shiotani A (2015). A traditional Japanese medicine–Hangeshashinto (TJ-14)–alleviates chemoradiation-induced mucositis and improves rates of treatment completion. Support Care Cancer.

[72] Ichiki M, Wataya H, Yamada K, Tsuruta N, Takeoka H, Okayama Y, Sasaki J, Hoshino T (2017). Preventive effect of kampo medicine (hangeshashin-to, TJ-14) plus minocycline against afatinib-induced diarrhea and skin rash in patients with non-small cell lung cancer. Onco Targets Ther.

[73] Mori K, Kondo T, Kamiyama Y, Kano Y, Tominaga K (2003). Preventive effect of Kampo medicine (Hangeshashin-to) against irinotecan-induced diarrhea in advanced non-small-cell lung cancer. Cancer Chemother Pharmacol.

[74] Matsuda C, Munemoto Y, Mishima H, Nagata N, Oshiro M, Kataoka M, Sakamoto J, Aoyama T, Morita S, Kono T (2015). Double-blind, placebo-controlled, randomized phase II study of TJ-14 (Hangeshashinto) for infusional fluorinated-pyrimidine-based colorectal cancer chemotherapy-induced oral mucositis. Cancer Chemother Pharmacol.

[75] Aoyama T, Nishikawa K, Takiguchi N, Tanabe K, Imano M, Fukushima R, Sakamoto J, Oba MS, Morita S, Kono T, Tsuburaya A (2014). Double-blind, placebo-controlled, randomized phase II study of TJ-14 (hangeshashinto) for gastric cancer chemotherapy-induced oral mucositis. Cancer Chemother Pharmacol.

[76] Nishikawa K, Aoyama T, Oba MS, Yoshikawa T, Matsuda C, Munemoto Y, Takiguchi N, Tanabe K, Nagata N, Imano M, Oshiro M, Fukushima R, Kataoka M, Morita S, Tsuburaya A, Mishima H, Kono T, Sakamoto J (2018). The clinical impact of Hangeshashinto (TJ-14) in the treatment of chemotherapy-induced oral mucositis in gastric cancer and colorectal cancer: analyses of pooled data from two phase II randomized clinical trials (HANGESHA-G and HANGESHA-C). J Cancer.

[77] Wu YL, Zhou C, Hu CP, Feng J, Lu S, Huang Y, Li W, Hou M, Shi JH, Lee KY, Xu CR, Massey D, Kim M, Shi Y, Geater SL (2014). Afatinib versus cisplatin plus gemcitabine for first-line treatment of Asian patients with advanced non-small-cell lung cancer harbouring EGFR mutations (LUX-Lung 6): an open-label, randomised phase 3 trial. Lancet Oncol.

[78] Sequist LV, Yang JC, Yamamoto N, O'Byrne K, Hirsh V, Mok T, Geater SL, Orlov S, Tsai CM, Boyer M, Su WC, Bennouna J, Kato T, Gorbunova V, Lee KH, Shah R, Massey D, Zazulina V, Shahidi M, Schuler M (2013). Phase III study of afatinib or cisplatin plus pemetrexed in patients with metastatic lung adenocarcinoma with EGFR mutations. J Clin Oncol.

[79] Vogler BK, Pittler MH, Ernst E (1999). The efficacy of ginseng. A systematic review of randomised clinical trials. Eur J Clin Pharmacol.

[80] Ahlberg K, Ekman T, Gaston-Johansson F, Mock V (2003). Assessment and management of cancer-related fatigue in adults. Lancet.

[81] Barton DL, Liu H, Dakhil SR, Linquist B, Sloan JA, Nichols CR, McGinn TW, Stella PJ, Seeger GR, Sood A, Loprinzi CL (2013). Wisconsin Ginseng (*Panax quinquefolius*) to improve cancer-related fatigue: a randomized, double-blind trial, N07C2. J Natl Cancer Inst.

[82] Jiang SL, Liu HJ, Liu ZC, Liu N, Liu R, Kang YR, Ji JG, Zhang C, Hua BJ, Kang SJ (2017). Adjuvant effects of fermented red ginseng extract on advanced non-small cell lung cancer patients treated with chemotherapy. Chin J Integr Med.

[83] Lam W, Bussom S, Guan F, Jiang Z, Zhang W, Gullen EA, Liu SH, Cheng YC (2010). The four-herb Chinese medicine PHY906 reduces chemotherapy-induced gastrointestinal toxicity. Sci Transl Med.

[84] Kummar S, Copur MS, Rose M, Wadler S, Stephenson J, O’Rourke M, Brenckman W, Tilton R, Liu SH, Jiang Z, Su T, Cheng YC, Chu E (2011). A phase I study of the chinese herbal medicine PHY906 as a modulator of irinotecan-based chemotherapy in patients with advanced colorectal cancer. Clin Colorectal Cancer.

[85] Changou CA, Shiah HS, Chen LT, Liu S, Luh F, Liu SH, Cheng YC, Yen Y (2021). A Phase II clinical trial on the combination therapy of PHY906 plus capecitabine in hepatocellular carcinoma. Oncologist.

[86] Sun TY, Li JS, Chen C (2015). Effects of blending wheatgrass juice on enhancing phenolic compounds and antioxidant activities of traditional kombucha beverage. J Food Drug Anal.

[87] Bar-Sela G, Tsalic M, Fried G, Goldberg H (2007). Wheat grass juice may improve hematological toxicity related to chemotherapy in breast cancer patients: a pilot study. Nutr Cancer.

[88] van Erp NP, Baker SD, Zandvliet AS, Ploeger BA, den Hollander M, Chen Z, den Hartigh J, König-Quartel JM, Guchelaar HJ, Gelderblom H (2011). Marginal increase of sunitinib exposure by grapefruit juice. Cancer Chemother Pharmacol.

[89] Gbolahan OB, O’Neil BH, McRee AJ, Sanoff HK, Fallon JK, Smith PC, Ivanova A, Moore DT, Dumond J, Asher GN (2022). A phase I evaluation of the effect of curcumin on dose-limiting toxicity and pharmacokinetics of irinotecan in participants with solid tumors. Clin Transl Sci.

[90] Braal CL, Hussaarts K, Seuren L, Oomen-de Hoop E, de Bruijn P, Buck SAJ, Bos M, Thijs-Visser MF, Zuetenhorst HJM, Mathijssen-van Stein D, Vastbinder MB, van Leeuwen RWF, van Gelder T, Koolen SLW, Jager A, Mathijssen RHJ (2020). Influence of green tea consumption on endoxifen steady-state concentration in breast cancer patients treated with tamoxifen. Breast Cancer Res Treat.

[91] Engels FK, de Jong FA, Sparreboom A, Mathot RA, Loos WJ, Kitzen JJ, de Bruijn P, Verweij J, Mathijssen RH (2007). Medicinal cannabis does not influence the clinical pharmacokinetics of irinotecan and docetaxel. Oncologist.

[92] Goey AK, Meijerman I, Rosing H, Burgers JA, Mergui-Roelvink M, Keessen M, Marchetti S, Beijnen JH, Schellens JH (2013). The effect of *Echinacea purpurea* on the pharmacokinetics of docetaxel. Br J Clin Pharmacol.

[93] Guha N, Kwan ML, Quesenberry CP, Weltzien EK, Castillo AL, Caan BJ (2009). Soy isoflavones and risk of cancer recurrence in a cohort of breast cancer survivors: the life after cancer epidemiology study. Breast Cancer Res Treat.

[94] Mansky PJ, Wallerstedt DB, Sannes TS, Stagl J, Johnson LL, Blackman MR, Grem JL, Swain SM, Monahan BP (2013). NCCAM/NCI Phase 1 study of mistletoe extract and gemcitabine in patients with advanced solid tumors. Evid Based Complement Alternat Med.

[95] Tobin PJ, Beale P, Noney L, Liddell S, Rivory LP, Clarke S (2006). A pilot study on the safety of combining chrysin, a non-absorbable inducer of UGT1A1, and irinotecan (CPT-11) to treat metastatic colorectal cancer. Cancer Chemother Pharmacol.

[96] Tocaciu S, Oliver LJ, Lowenthal RM, Peterson GM, Patel R, Shastri M, McGuinness G, Olesen I, Fitton JH (2018). The effect of undaria pinnatifida fucoidan on the pharmacokinetics of letrozole and tamoxifen in patients with breast cancer. Integr Cancer Ther.

[97] van Erp NP, Baker SD, Zhao M, Rudek MA, Guchelaar HJ, Nortier JW, Sparreboom A, Gelderblom H (2005). Effect of milk thistle (*Silybum marianum*) on the pharmacokinetics of irinotecan. Clin Cancer Res.

[98] Vardy J, Dhillon HM, Clarke SJ, Olesen I, Leslie F, Warby A, Beith J, Sullivan A, Hamilton A, Beale P, Rittau A, McLachlan AJ (2013). Investigation of herb-drug interactions with ginkgo biloba in women receiving hormonal treatment for early breast cancer. Springerplus.

[99] Veerman GDM, van der Werff SC, Koolen SLW, Miedema JR, Oomen-de Hoop E, van der Mark SC, Chandoesing PP, de Bruijn P, Wijsenbeek MS, Mathijssen RHJ (2022). The influence of green tea extract on nintedanib’s bioavailability in patients with pulmonary fibrosis. Biomed Pharmacother.

