# Supplementary material for: Interactions between natural products and cancer treatments: underlying mechanisms and clinical importance
Source: Cancer Chemother Pharmacol. 2023 Jan 27;91(2):103–19. doi: 10.1007/s00280-023-04504-z (PMC9905199; doi:10.1007/s00280-023-04504-z)
Supplement: Supplementary file 1 — Supplementary file1 (DOCX 17 KB) [file 280_2023_4504_MOESM1_ESM.docx]

| Search Pubmed |
| --- |
| **Top-Selling Herbal Supplements in 2020** |
| Elder berry OR Sambucus nigra OR Sambucus canadensis OR Horehound OR Marrubium vulgare OR cranberry OR Vaccinium macrocarpon OR turmeric OR curcumin OR Curcuma longa OR Apple cider vinegar OR malus spp. OR ginger OR Zingiber officinale OR Echinacea OR Echinacea spp. OR garlic OR Allium sativum OR Fenugreek OR Trigonella foenum-graecum OR Wheatgrass OR Barley grass OR Triticum aestivum OR Hordeum vulgare OR Saw palmetto OR Serenoa repens OR Ashwagandha OR Withania somnifera OR Green tea OR Camellia sinensis OR Ivy leaf OR Hedera helix OR Ginkgo OR Ginkgo biloba OR Cannabis OR Cannabidiol OR CBD OR Cannabis sativa OR Black cohosh OR Actaea racemose OR Beta-sitosterol OR Red yeast rice OR Oryza sativa OR Aloe OR Aloe vera OR St John’s wort OR Hypericum perforatum OR Flax seed / Flax oil OR Linum usitatissimum OR Milk thistle OR Silybum marianum OR Yohimbe OR Pausinystalia johimbe OR Corynanthe johimbe OR Goji berry OR Lycium spp OR Valerian OR Valeriana officinalis OR Horny goat weed OR Epimedium spp OR Bioflavonoid complex OR Beet root OR Beta vulgaris OR Cinnamon OR Cinnamomum spp. OR Senna OR Senna alexandrina OR Green coffee extract OR Coffea arabica OR Plant sterols OR Ginseng OR Panax spp. OR Chamomile OR Matricaria chamomilla OR Matricaria recutita OR Garcinia OR Garcinia gummi-gutta OR Fennel OR Foeniculum vulgare OR Maca OR Lepidium meyenii OR Açaí OR Euterpe oleracea OR Rhodiola OR Rhodiola spp. OR grapefruit OR grapefruit juice OR Schisandra OR Wuzhi OR TJ-14 OR hangeshashinto OR PHY906 OR soy OR soy food OR isoflavones |
| **Interaction phrases** |
| Chemotherapy OR anti-cancer OR anticancer drug OR interaction OR Drug-herb interaction OR Drug-drug interaction OR herb-drug interaction OR Pharmacokinetic* OR bioavailability OR absorption OR distribution OR metabolism OR elimination OR Pharmacodynamic* OR pharmacokinetic interaction* OR pharmacodynamic interaction* |
| **Essential medicines for cancer on the national essential medicines lists or national reimbursable medicines lists of 135 countries, 2015** |
| Asparaginase OR Bleomycin OR Calcium folinate OR Carboplatin OR Chlorambucil OR Cyclophosphamide OR Cytarabine OR Dacarbazine OR Dactinomycin OR Daunorubicin OR Docetaxel OR Doxorubicin OR Etoposide OR Fluorouracil OR Hydroxycarbamide OR Ifosfamide OR Mercaptopurine OR Mesna OR Methotrexate OR Paclitaxel OR Procarbazine OR Tamoxifen OR Tioguanine OR Vinblastine OR Vincristine OR tretinoin OR Anastrazole OR exemestane OR letrozole OR Bendamustine OR Bicalutamide OR Capecitabine OR Cisplatin OR Fludarabine OR Gemcitabine OR Filgrastim OR lenograstim OR pegfilgrastim OR Imatinib OR Irinotecan OR Goserelin OR leuprolin OR triptorelin OR Oxaliplatin OR Rituximab OR Trastuzumab OR Vinorelbine OR Arsenic trioxide OR Dasatinib OR Diethylstilboestrol OR Erlotinib OR Gefitinib OR Nilotinib |
| **Results – Abstract/ Full text/ clinical study/ clinical trials/ English/ from 2000** |
| 7,545 |
| Results: 22 papers identified – plus papers identified in cancer resources and bibliographies of the 22 papers = 17.  Total papers included 39 |
