# Supplementary material for: Interactions between natural products and cancer treatments: underlying mechanisms and clinical importance
Source: Cancer Chemother Pharmacol. 2023 Jan 27;91(2):103–19. doi: 10.1007/s00280-023-04504-z (PMC9905199; doi:10.1007/s00280-023-04504-z)

Herbal Supplement Sales in US Increase by Record-Breaking 17.3% in 2020

Sales of immune health, stress relief, and heart health supplements grow during COVID-19 pandemic

By Tyler Smith,[a] Farhana Majid,[b] Veronica Eckl,[b] and Claire Morton Reynolds[c]

[a] American Botanical Council; Austin, Texas
[b] SPINS; Chicago, Illinois
[c] *Nutrition Business Journal*; Boulder, Colorado

Introduction

Herbal dietary supplement sales in the United States surpassed $10 billion for the first time in 2020, totaling an estimated $11.261 billion, according to the *Nutrition Business Journal* (NBJ). In 2020, sales of these products, which include dietary supplements with herbal and/or fungal ingredients, increased by a record-shattering 17.3% from 2019 — the first time this segment has experienced double-digit growth in at least the past two decades (Table 1). Previously, since 2000, the highest percentage sales increase for herbal supplements was 9.4%, from 2017 to 2018. Consumers in the United States spent $1.659 billion more on herbal supplements in 2020 than in 2019, which is more than the annual spending increases from 2017 to 2018 and 2018 to 2019 combined.

SPINS, a market research firm based in Chicago, Illinois, and NBJ, a natural products industry publication of Informa's New Hope Network based in Boulder, Colorado, provided the US retail sales figures for this report. NBJ supplied estimates of the total annual sales of herbal supplements, as well as sales in three market channels (mass market; natural, health food, and specialty; and direct sales) and sales by product type (single-herb supplements vs. combination formulas). SPINS provided sales data for the 40 top-selling herbal and fungal ingredients in the mainstream (conventional) and natural retail channels. Channel definitions are included in Table 2.

Table 1. Total US Retail Sales of Herbal Supplements*

Year	Total Sales (in Billions)	% Change
**2020**	$11.261	17.3%
**2019**	$9.602	8.6%
**2018**	$8.842	9.4%
**2017**	$8.085	8.5%
**2016**	$7.452	7.7%
**2015**	$6.922	7.5%
**2014**	$6.441	6.8%
**2013**	$6.033	7.9%
**2012**	$5.593	5.5%
**2011**	$5.302	4.5%
**2010**	$5.049	3.3%
**2009**	$5.037	5.0%
**2008**	$4.800	1.0%
**2007**	$4.756	4.4%
**2006**	$4.558	4.1%
**2005**	$4.378	2.1%
**2004**	$4.288	3.4%
**2003**	$4.146	–2.3%
**2002**	$4.275	–2.8%
**2001**	$4.361	3.2%
**2000**	$4.225	2.9%

Source: *Nutrition Business Journal*

* Includes sales in all channels. NBJ primary research includes NBJ surveys of supplement manufacturers, distributors, MLM firms, mail order, internet, and raw material and ingredient supply companies, as well as numerous interviews with major retailers (Walmart, Costco, etc.), manufacturers, suppliers, and industry experts. Secondary sources include IRI, SPINS Natural, Nielsen, *Natural Foods Merchandiser*, Insight, The Hartman Group, company data, and other published material.

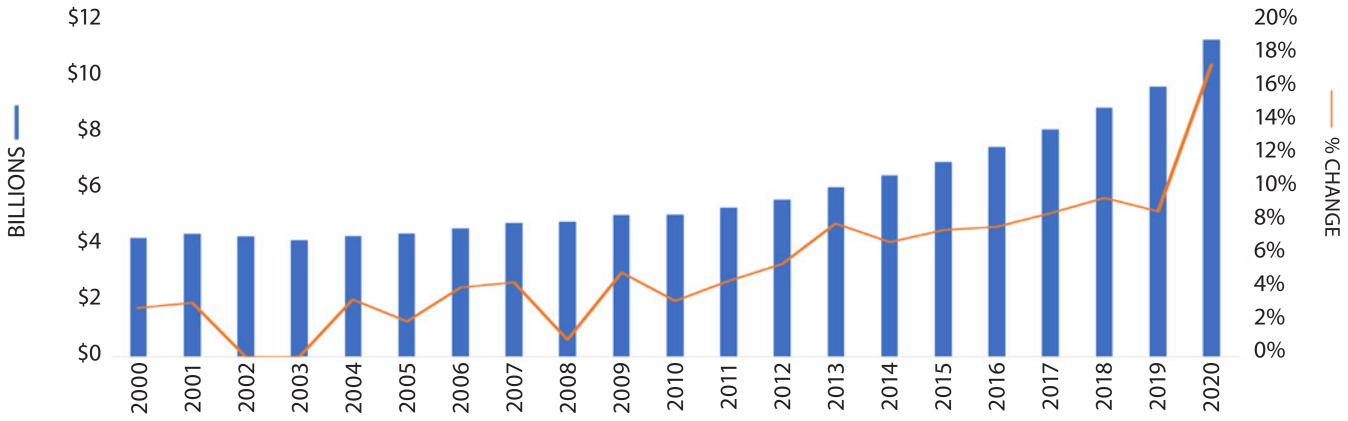

Table 2. US Retail Channel Definitions*		
	SPINS	*Nutrition Business Journal*
**Mainstream Retail Channels**	**MultiOutlet Channel (powered by IRI)**<br>Covers grocery outlets (stores with $2 million+ total annual sales), drug outlets (chains and independent stores, excluding prescription sales), and selected retailers across mass merchandisers, including Walmart, club, dollar, and military stores representing more than 105,000 retail locations.	**Mass Market Channel**<br>Includes food/grocery, drug, mass merchandise, and club and convenience stores, including Walmart, Costco, etc.
**Natural Retail Channels**	**Natural Enhanced Channel**<br>Includes full-format stores with $2 million+ in annual sales and 40% or more of UPC-coded sales from natural/organic/specialty products. It includes co-ops, associations, independents, and large regional chains (excluding Whole Foods Market & Trader Joe's). This channel breeds innovation and sustains the level of authenticity and high product standards that define the industry. It represents more than $28 billion in total sales, and encompasses more than 1,850 stores.	**Natural, Health Food, and Specialty Channel**<br>Includes supplement and specialty retail outlets, including Whole Foods Market (estimates), GNC, sports nutrition stores, etc.
**Direct Sales**		Includes direct-to-consumer sales from the internet (e.g., e-commerce websites such as Amazon.com and Walmart.com, among many others), direct-selling media (TV, radio, and print publications), health practitioners, and multilevel marketing (MLM) or network marketing firms (US sales only).

* The sales discussed in this article pertain only to those involving herbal or fungal dietary supplements, and generally do not include herbs sold as teas and beverages or as ingredients in natural personal care and cosmetic products, including so-called "cosmeceutical" products.

In 2020, for the 12th consecutive year, sales increased in each of NBJ's market channels (Table 3). The strongest growth in 2020 was in the mass market channel, which increased by 25.1% from 2019 and totaled $2.131 billion in 2020. This is more than double the sales growth of 9.4% in this channel from 2018 to 2019. Direct sales of herbal supplements, which include online sales, increased by 23.7% in 2020 — more than twice the percentage growth of 11.5% seen in 2019. Sales in NBJ's natural, health food, and specialty channel increased by 1.6% in 2020 and totaled $2.950 billion. Despite the moderate sales growth in NBJ's natural channel, total sales in this channel have been higher than in the mass market channel since at least 2005, when *HerbalGram* began including sales data for the three NBJ market channels. In recent years, however, the difference in total sales between these two channels has decreased.

The SPINS data for the ingredients discussed in this report include sales of dietary supplements in which the herbal or fungal ingredient (or derivative thereof, such as plant sterols, quercetin, etc.) is the primary functional ingredient. This includes only products that meet the legal definition of a dietary supplement per the US Food and Drug Administration (FDA), except for cannabidiol (CBD) products, as explained later.[1] Sales of herbal teas or cosmetics with botanical ingredients are not included. The dollar amounts reflect the latest estimates (as of late June 2021) of sales for the 52-week period that ended December 27, 2020.

Table 3. Total Herbal Supplement Sales in US by Channel (in Billions)							
	2015	2016	2017	2018	2019	2020	% Growth*
**Mass Market**	$1.204	$1.336	$1.449	$1.558	$1.704	$2.131	25.1%
**Natural, Health Food, and Specialty**	$2.356	$2.506	$2.624	$2.804	$2.904	$2.950	1.6%
**Direct Sales**	$3.363	$3.609	$4.012	$4.480	$4.995	$6.179	23.7%

Source: *Nutrition Business Journal*
* From 2019

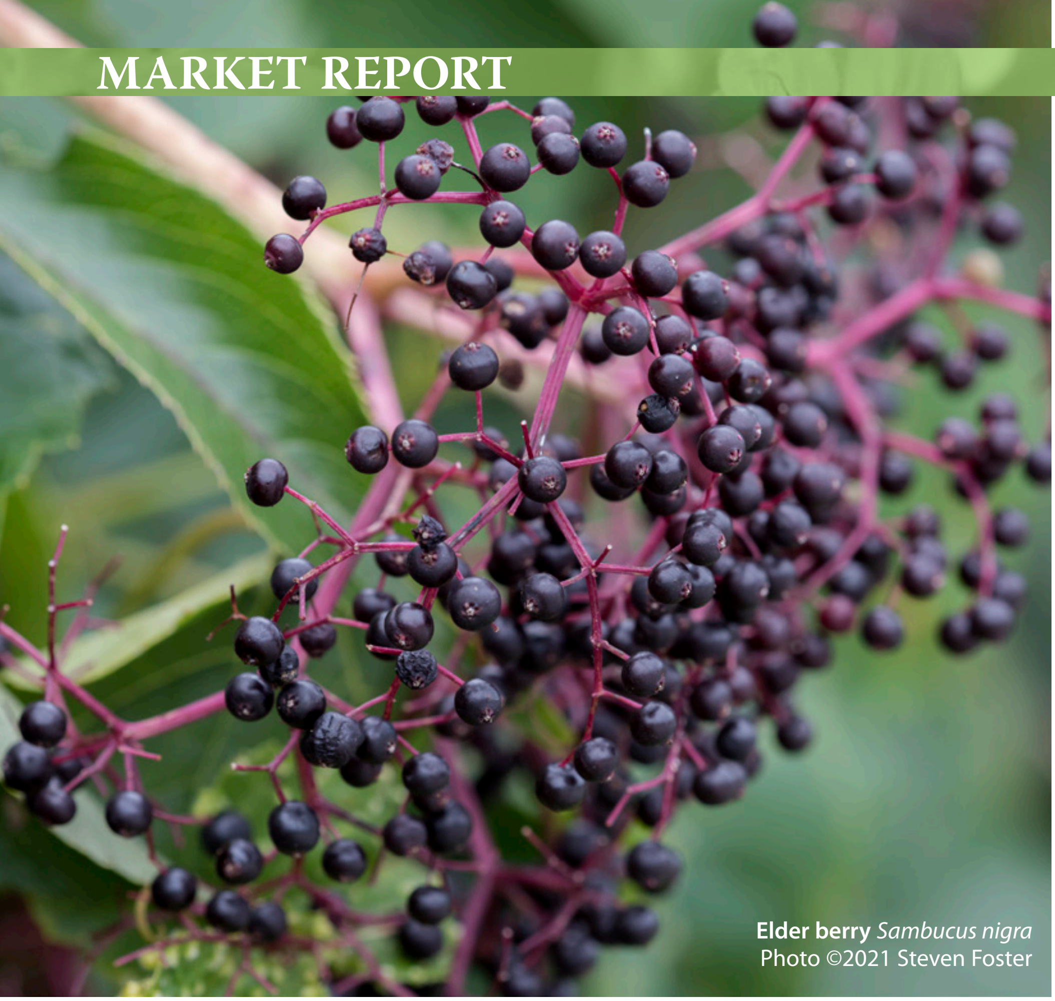

Elder berry *Sambucus nigra*
Photo ©2021 Steven Foster

Unless otherwise noted, subsequent descriptions of sales increases and decreases, or sales growth and decline, refer to total annual sales changes *by percentage* from the previous year. The mainstream and natural channel sales discussed in this report refer to retail sales in the United States only.

Elder Berry, Ashwagandha, and Apple Cider Vinegar Supplement Sales Drive Growth in Mainstream Channel

Elder Berry

In 2020, elder berry (*Sambucus* spp., Viburnaceae), frequently written as "elderberry," was the top-selling herbal dietary supplement ingredient in mainstream retail outlets. Consumers spent an estimated $275,544,691 on elder berry supplements in this channel in 2020, an increase of 150.3% from 2019, according to SPINS. Elder berry sales in this channel more than doubled each year from 2018 to 2020, and consistently increasing sales moved the herb from the 25th top-selling ingredient in 2015 to the top spot in 2020. Elder berry displaced horehound (*Marrubium vulgare*, Lamiaceae), which was the top-selling herbal ingredient in the mainstream channel from 2013 to 2019. Horehound is traditionally used for respiratory conditions[2] and is a common ingredient in several well-known brands of throat lozenges, including Ricola® (Ricola Ltd.; Laufen, Switzerland).

Elder berry is commonly used to support immune health and as a remedy to help alleviate cold and flu symptoms, and these potential benefits have been investigated in human clinical trials. A systematic review of elder berry for the prevention and treatment of viral respiratory illnesses, published in April 2021, found that elder berry may reduce the severity and duration of colds, and the duration of the flu. Although the authors rated much of the evidence as uncertain, they concluded that "elderberry may be a safe option for treating viral respiratory illness."[3] Similarly, a 2019 meta-analysis concluded that the herb "substantially reduce[d] upper respiratory symptoms."[4]

According to the 2020 Council for Responsible Nutrition (CRN) Consumer Survey on Dietary Supplements, immune health was the second most common reason why US consumers took supplements in 2020. In the 18-34 age group, immune health was the top reason.[5]

Google searches for "elderberry" peaked in late March 2020, shortly after the World Health Organization declared the COVID-19 outbreak a pandemic,[6] and the fifth most-common search with "elderberry" in 2020 was "elderberry coronavirus."[7] According to the US National Center for Complementary and Integrative Health (NCCIH), there were no published human clinical trials related to elder berry and COVID-19 as of July 2021,[8] and the FDA sent warning letters to at least five companies in 2020 for marketing products with unsubstantiated claims involving elder berry and COVID-19.[9]

As of July 2021, at least two human studies involving elder berry and COVID-19 were in progress outside of the United States. One randomized, placebo-controlled clinical trial, sponsored by Mashhad University of Medical Sciences in Mashhad, Iran, will assess the effects of an investigator-prepared elder berry extract syrup on COVID-19 symptoms in outpatients and patients quarantined at home.[10] The second, led by researchers at East Kent Hospitals University/National Health Service Foundation Trust in England, is recruiting participants for a study examining the effects of Sambucol® Black Elderberry syrup (PharmaCare US, Inc.; San Diego, CA) on the "treatment, progression, and reduction of symptoms" in patients with COVID-19.[11]

According to CRN's consumer survey, elder berry ranked among the top 10 ingredients US consumers took to support their immune health in 2020.[12] Echinacea (*Echinacea* spp., Asteraceae), garlic (*Allium sativum*, Amaryllidaceae), and turmeric (*Curcuma longa*, Zingiberaceae) also made the top 10 list of immune ingredients, and each of these herbs experienced sales increases in the 2020 mainstream retail channel. Of these, echinacea experienced the strongest sales growth of 36.8%.

Ashwagandha

Ashwagandha (*Withania somnifera*, Solanaceae) experienced the greatest sales growth in the mainstream channel, with sales increasing by 185.2% to a total of $31,742,304 in 2020. Ashwagandha first appeared among the 40 top-selling herbs in the mainstream retail channel in 2018, when it was ranked 34th in sales. Since then, as many mainstream consumers have become more familiar with the herb, annual sales have more than quadrupled, and ashwagandha was the 12th top-selling herb in 2020.

Widely used in Ayurveda, the primary traditional medical system of India, ashwagandha is a well-known adaptogen, a term generally used to describe a substance that increases the body's ability to resist, or adapt to, stress.[13,14] In Ayurvedic medicine, ashwagandha is known as a *rasayana*, or rejuvenator, and is used as a "nourishing agent for fatigue and deficiency of *prana* (the life vital energy … in Ayurveda)."[15] In Sanskrit, *ashwa* means "horse" and *gandha* means "smell," and the root is said to have the smell of a horse and give those who consume it the power of a horse. It has been used traditionally as a tonic to strengthen organ systems and as an aphrodisiac, stimulant, and thermogenic (metabolism stimulator), among other uses.[13]

Pharmacological studies have found that ashwagandha has anti-inflammatory, neuroprotective, sleep-inducing, and anxiolytic properties.[16] Modern research also has examined ashwagandha's stimulating and stress-relieving properties. A systematic review published in 2021 concluded that ashwagandha was more effective than placebo in improving various physical performance variables.[17] A separate systematic review published in 2021 assessed the effects of more than a dozen single plants or phytocompounds on the hypothalamic-pituitary-adrenal (HPA) axis, which plays a central role in the body's stress response. They concluded that the evidence for many of the plants was unclear, but "the most consistent finding was a morning, cortisol-lowering effect from ashwagandha supplementation."[18] (Cortisol is a hormone with many physiological functions. Most notably, it reduces inflammation, increases blood sugar, and helps the body respond to stress.[19])

According to CRN's 2020 COVID-19 Consumer Survey, 43% of supplement users changed their supplement routines since the beginning of the pandemic, and of those, 91% increased their supplement intake. When asked why these consumers increased their supplement intake, nearly a quarter cited mental health reasons, including stress and anxiety.[20]

Apple Cider Vinegar

Apple (*Malus* spp., Rosaceae) cider vinegar (ACV) was the only other herbal supplement ingredient in the mainstream channel with a sales increase of more than 100% from 2019. Consumers spent $79,257,715 on ACV supplements in 2020, a 133.8% increase from 2019. ACV first appeared on the list of 40 top-selling herbal supplements in the mainstream channel in 2019 after a 10.4% sales increase from the previous year. Significantly increased

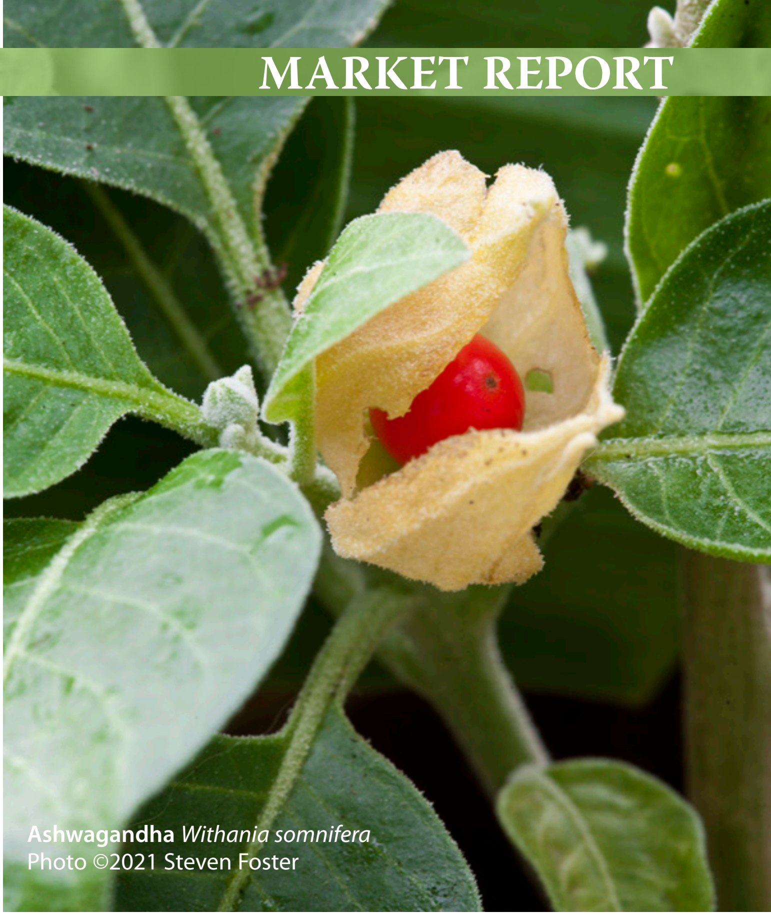

Ashwagandha *Withania somnifera*
Photo ©2021 Steven Foster

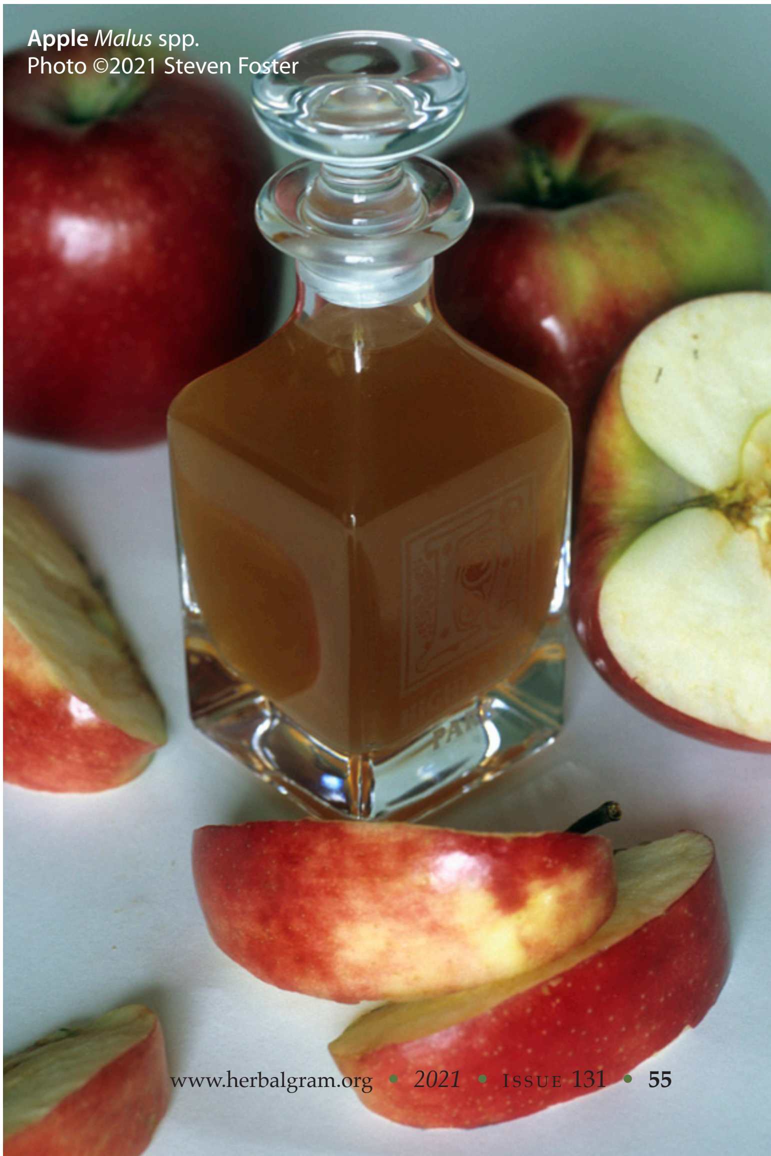

Apple *Malus* spp.
Photo ©2021 Steven Foster

Table 4. Top-Selling Herbal Supplements in 2020 — US Mainstream Multi-Outlet Channel				
**Rank**	**Primary Ingredient**	**Latin Binomial**	**Total Sales**	**% Change from 2019**
1	Elder berry	*Sambucus nigra* and *S. canadensis*	$275,544,691	150.3%
2	Horehound	*Marrubium vulgare*	$137,054,571	–11.4%
3	Cranberry	*Vaccinium macrocarpon*	$101,339,826	12.9%
4	Turmeric[a]	*Curcuma longa*	$96,971,371	3.1%
5	Apple cider vinegar	*Malus* spp.	$79,257,715	133.8%
6	Ginger	*Zingiber officinale*	$64,779,632	39.3%
7	Echinacea[b]	*Echinacea* spp.	$57,345,210	36.8%
8	Garlic	*Allium sativum*	$42,924,030	12.1%
9	Fenugreek	*Trigonella foenum-graecum*	$35,148,440	5.5%
10	Wheatgrass / Barley grass	*Triticum aestivum / Hordeum vulgare*	$32,887,254	9.2%
11	Saw palmetto	*Serenoa repens*	$32,697,628	5.4%
12	Ashwagandha	*Withania somnifera*	$31,742,304	185.2%
13	Green tea	*Camellia sinensis*	$31,408,078	–7.9%
14	Ivy leaf	*Hedera helix*	$29,581,801	–32.0%
15	Ginkgo	*Ginkgo biloba*	$28,576,480	9.7%
16	Cannabidiol (CBD)	*Cannabis sativa*	$26,551,872	–30.0%
17	Black cohosh	*Actaea racemosa*	$24,890,605	–12.0%
18	Beta-sitosterol[c]	—	$24,827,065	52.3%
19	Red yeast rice[d]	*Oryza sativa*	$24,613,191	–3.9%
20	Aloe	*Aloe vera*	$24,403,736	11.2%
21	St John's wort	*Hypericum perforatum*	$23,890,515	0.3%
22	Flax seed / Flax oil	*Linum usitatissimum*	$22,150,127	–3.2%
23	Milk thistle	*Silybum marianum*	$19,823,644	8.4%
24	Yohimbe	*Pausinystalia johimbe* syn. *Corynanthe johimbe*	$17,774,381	–3.7%
25	Goji berry	*Lycium* spp.	$16,104,457	15.9%
26	Valerian	*Valeriana officinalis*	$14,596,855	–11.2%
27	Horny goat weed	*Epimedium* spp.	$14,546,366	1.6%
28	Bioflavonoid complex[e]	—	$14,137,366	–4.2%
29	Beet root	*Beta vulgaris*	$13,945,332	22.4%
30	Cinnamon	*Cinnamomum* spp.	$12,339,671	–18.2%
31	Senna[f]	*Senna alexandrina*	$12,295,396	1.0%
32	Green coffee extract	*Coffea arabica*	$12,263,598	–21.1%
33	Plant sterols[g]	—	$11,498,813	4.3%
34	Ginseng	*Panax* spp.	$11,200,292	–11.9%
35	Chamomile	*Matricaria chamomilla* syn. *M. recutita*	$10,624,567	30.1%
36	Garcinia	*Garcinia gummi-gutta*	$10,618,783	–35.7%
37	Fennel	*Foeniculum vulgare*	$10,101,137	9.2%
38	Maca	*Lepidium meyenii*	$10,075,136	21.8%
39	Açaí	*Euterpe oleracea*	$9,835,442	10.4%
40	Rhodiola	*Rhodiola* spp.	$8,433,070	–4.3%

Source: SPINS (52 weeks ending December 27, 2020)

[a] Includes standardized turmeric extracts with high levels of curcumin.
[b] Includes three *Echinacea* species: *E. angustifolia*, *E. pallida*, and *E. purpurea*.
[c] Beta-sitosterol is a common plant sterol that can be derived from various plants.
[d] Red yeast rice is fermented with the yeast *Monascus purpureus*.
[e] Bioflavonoids are phytochemicals that are often extracted from citrus (*Citrus* spp.) fruits.
[f] Excludes over-the-counter laxative drugs containing senna or sennosides.
[g] Not including beta-sitosterol.

Table 5. Top-Selling Herbal Supplements in 2020 — US Natural Channel

Rank	Primary Ingredient	Latin Binomial	Total Sales	% Change from 2019
1	Cannabidiol (CBD)	*Cannabis sativa*	$57,217,025	–36.9%
2	Elder berry	*Sambucus nigra* and *S. canadensis*	$54,132,170	68.2%
3	Turmeric[a]	*Curcuma longa*	$41,457,226	–13.7%
4	Wheatgrass / Barley grass	*Triticum aestivum / Hordeum vulgare*	$17,484,031	–3.0%
5	Mushrooms (other)	—	$14,986,621	41.8%
6	Aloe	*Aloe vera*	$13,784,172	10.2%
7	Ashwagandha	*Withania somnifera*	$13,507,542	–1.2%
8	Oregano[b]	*Origanum vulgare*	$13,327,549	43.2%
9	Echinacea[c]	*Echinacea* spp.	$12,856,524	23.8%
10	Flax seed / Flax oil	*Linum usitatissimum*	$11,522,131	–8.9%
11	Milk thistle	*Silybum marianum*	$9,152,946	–8.4%
12	Echinacea–goldenseal combo	*Echinacea* spp. / *Hydrastis canadensis*	$7,991,152	27.3%
13	Saw palmetto	*Serenoa repens*	$7,813,043	0.8%
14	Cranberry	*Vaccinium macrocarpon*	$7,715,667	–3.1%
15	Garlic	*Allium sativum*	$7,559,525	10.5%
16	Maca	*Lepidium meyenii*	$7,105,435	–5.8%
17	Valerian	*Valeriana officinalis*	$6,818,876	0.4%
18	Nigella	*Nigella sativa*	$6,468,066	21.6%
19	Quercetin[d]	—	$6,415,921	74.1%
20	Chlorophyll / Chlorella	— / *Chlorella vulgaris*	$5,258,353	1.6%
21	Ginkgo	*Ginkgo biloba*	$4,491,630	–1.8%
22	Horsetail	*Equisetum* spp.	$4,490,279	–16.0%
23	Reishi mushrooms	*Ganoderma lucidum*	$4,480,026	22.7%
24	Beet root	*Beta vulgaris*	$3,691,197	18.0%
25	Apple cider vinegar	*Malus* spp.	$3,609,865	97.7%
26	Olive leaf	*Olea europaea*	$3,580,845	24.0%
27	Red yeast rice[e]	*Oryza sativa*	$3,518,993	–6.5%
28	Cordyceps mushrooms	*Cordyceps* spp.	$3,482,578	25.7%
29	Kava	*Piper methysticum*	$3,414,784	0.1%
30	Ginseng	*Panax* spp.	$3,253,793	–1.2%
31	Cherry	*Prunus* spp.	$3,245,910	–11.3%
32	Ginger	*Zingiber officinale*	$3,197,474	–4.1%
33	Resveratrol[f]	—	$3,021,575	–0.1%
34	Fenugreek	*Trigonella foenum-graecum*	$2,981,690	–9.3%
35	Holy basil	*Ocimum tenuiflorum*	$2,851,773	–7.9%
36	Hawthorn	*Crataegus* spp.	$2,816,112	–3.8%
37	Stevia	*Stevia rebaudiana*	$2,738,754	–9.7%
38	Evening primrose oil	*Oenothera biennis*	$2,738,213	–8.1%
39	Nettle	*Urtica dioica*	$2,731,865	0.6%
40	Chaga mushrooms	*Inonotus obliquus*	$2,730,506	54.9%

Source: SPINS (52 weeks ending December 27, 2020)

[a] Includes standardized turmeric extracts with high levels of curcumin.
[b] Includes products labeled as containing oregano oil and oregano leaf tinctures.
[c] Includes three *Echinacea* species: *E. angustifolia*, *E. pallida*, and *E. purpurea*.
[d] Quercetin is a flavonoid found in various plants, such as onions and berries.
[e] Red yeast rice is fermented with the yeast *Monascus purpureus*.
[f] Resveratrol is an antioxidant found in various plants, such as grapes and berries.

ACV has been promoted for weight loss, regulation of blood sugar and blood pressure, digestive health, cholesterol reduction, immune support, and skin care, among many other uses.

mainstream sales in 2020 moved it from the 12th top-selling supplement in this channel in 2019 to the fifth in 2020. ACV supplements also had strong sales in natural retail outlets, as discussed later, and experienced the greatest percentage sales increase of any of the top 40 natural channel ingredients.

ACV is created when apples undergo a double fermentation process. First, crushed apples are soaked in water, and yeast converts the natural sugars in the apples to alcohol (alcoholic fermentation). Then, alcohol is converted to acetic acid, the main component of vinegar, in the presence of bacteria (acetic fermentation). Over time, the liquid will turn murky, and this cloud of yeast and bacteria is known as the "mother." Some people credit the mother, which is considered a probiotic, with the potential health benefits of ACV.[21,22]

ACV has been used as a food, medicine, and preserving or pickling agent since ancient times.[23] In the 20th century, physician D. C. Jarvis, MD, recommended it as a "cure-all" in his 1958 book *Folk Medicine: A Vermont Doctor's Guide to Good Health* (Henry Holt), which helped popularize the vinegar in US folk medicine.[24] In the 1980s, renowned herbalist Rosemary Gladstar included an ACV-based herbal formula known as Fire Cider in the first edition of her home study course, *The Science and Art of Herbalism*. Fire Cider is made by soaking various "fiery" herbs such as garlic and ginger (*Zingiber officinale*, Zingiberaceae) in ACV over a period of weeks, and it remains a popular herbal tonic.[25] Today, ACV is commonly found in grocery stores and used in vinaigrettes, marinades, and for other culinary purposes.

Although ACV has been a staple pantry ingredient and popular home remedy for decades, its popularity has increased in recent years, as major media outlets, health blogs, social media influencers, and celebrities tout its potential health benefits.[26,27] ACV has been promoted for weight loss, regulation of blood sugar and blood pressure, digestive health, cholesterol reduction, immune support, and skin care, among many other uses.[21,23] According to SPINS, mainstream ACV supplements marketed for "cleanse and detox" and digestive health had the greatest overall sales increases in 2020.

New, more palatable formulation types may have also contributed to increased ACV sales in 2020. ACV supplements are available in capsules, tablets, and powders, and, more recently, gummies.[26] Of the top-10 rising Google searches involving "apple cider vinegar" in 2020, eight included a reference to gummies.[28] Goli®, a West Hollywood, California-based supplement company launched what they call the "world's first" ACV gummy in September 2019,[29] and it achieved an almost "cult-like status" in 2020 among health bloggers, influencers, and others.[30] On its website, Goli mentions that the product may help support immune health, cellular energy production, heart health, healthy nutrient metabolism, a healthy nervous system, and overall good health. Goli's television ads featuring celebrity Jennifer Lopez likely helped increase public awareness of ACV's potential benefits and boost sales in 2020.

Despite the widespread health claims, human clinical research on ACV is limited. Still, a number of small studies have reported positive findings, particularly related to glycemic control and lipid profiles. In a 2019 systematic review and meta-analysis of the health effects of vinegar, the authors reported that consumption of ACV was associated with better glycemic control in patients with type 2 diabetes compared to other vinegars.[31,32] A systematic review and meta-analysis published in 2021 examined the effects of ACV on lipid profiles and glycemic parameters and concluded that ACV had significant, positive effects on blood sugar and blood lipid levels.[33] High-quality research on ACV's effects on metabolism and weight loss is lacking, according to a separate systematic review published in 2020.[34]

Beta-sitosterol

Beta-sitosterol supplements had the fourth highest sales increase in the mainstream channel in 2020. Consumers spent $24,827,065 on beta-sitosterol supplements in mainstream retail outlets in 2020, a 52.3% increase in total sales from the previous year. Beta-sitosterol is one of the most abundant plant sterols (also known as phytosterols) in the human diet,[35] and this is the first time it has appeared on the list of the 40 top-selling mainstream herbal supplements. SPINS has a separate category for other (non-beta-sitosterol) plant sterols, which have been among the top 40 mainstream ingredients since 2013 (except for 2018).

Beta-sitosterol has a chemical structure similar to cholesterol.[36] It is found in a wide variety of plants, including rice (*Oryza sativa*, Poaceae), wheat (*Triticum aestivum*, Poaceae), soy (*Glycine max*, Fabaceae), and peanuts (*Arachis hypogaea*, Fabaceae), among others.[37] As a dietary supplement ingredient, it is commonly marketed for benign prostatic hyperplasia (BPH), also known as enlarged prostate, as well as cardiovascular health and cholesterol management.[38] According to SPINS, beta-sitosterol supplements marketed for prostate health made up the majority of overall mainstream sales of beta-sitosterol in 2020, followed by cardiovascular health and immune health.

Although some human clinical trials have focused on the potential health effects of beta-sitosterol specifically, many studies and reviews focus on plant sterols in general. A systematic review published in 2000 found that beta-sitosterol was able to significantly improve urinary symptoms

and flow in men with BPH, a condition that affects up to 70% of men in their 60s and up to 80% of men 70 years of age and older.[39,40] Multiple meta-analyses have examined the effects of plant sterols on low-density lipoprotein (LDL) cholesterol ("bad cholesterol"), and, in general, researchers have found that plant sterols decrease LDL cholesterol levels in a dose-dependent manner.[41] A 2019 meta-analysis found that plant sterol supplementation correlated with reduced systolic and diastolic blood pressure.[42]

Increased mainstream sales of beta-sitosterol supplements in 2020 may be due in part to increased focus on what some consider age-related health concerns, such as cardiovascular health and prostate health. Interest in age-related products for men also has been steadily growing in recent years.[43] From 2010 to 2020, the 65+ population in the United States grew by more than a third, an increase corresponding to roughly 12.8 million people.[44] According to CRN, 81% of surveyed individuals 55 years of age and older took dietary supplements in 2020, more than any other age group.[5]

Three other ingredients had sales increases greater than 30% in the 2020 mainstream channel: ginger (39.3%), an herb commonly used for nausea, vomiting, and digestive issues[45]; echinacea (36.8%), one of CRN's top 10 ingredients for immunity in 2020[5]; and chamomile (*Matricaria chamomilla* syn. *M. recutita*, Asteraceae; 30.1%), an herb with calming properties.[46]

Mainstream Sales Decreases

Three herbs in the 2020 mainstream channel had sales decreases of 30% or more: garcinia (*Garcinia gummi-gutta* syn. *G. cambogia*, Clusiaceae; 35.7%), ivy leaf (*Hedera helix*, Araliaceae; 32.0%), and CBD (30.0%), which will be discussed in the next section.

Garcinia is a Southeast Asian fruit commonly marketed for weight loss. In 2020, mainstream garcinia sales totaled $10,618,783, a 35.7% decrease from 2019. After Mehmet Oz, MD, promoted the herb as a "magic weight loss cure" on his daytime television talk show, "The Dr. Oz Show," in 2012, garcinia sales skyrocketed. In 2015, at the height of its popularity, mainstream consumers spent more than $54 million on garcinia supplements, roughly five times more than was spent in 2020. Mainstream sales have steadily declined since 2015, after several high-profile legal challenges related to garcinia products, including lawsuits, a US Senate hearing, and a Federal Trade Commission settlement with a garcinia marketer that bought fake Amazon reviews.[47] Since then, the ingredient went from the fourth top-selling herb in this channel to the 36th in 2020.

Mainstream sales of ivy leaf supplements totaled $29,581,801 in 2020, a 32.0% decline from the previous year. This is the first time that ivy leaf sales have dropped since 2014, when it first appeared on the mainstream top 40 list. Ivy leaf is used traditionally (primarily in Europe) for cough and viral respiratory infections, such as the cold, flu, and bronchitis.[48] According to SPINS, most of the ivy leaf products included in the sales data are marketed for children and are generally used post-infection. Decreased mainstream sales in 2020 may be due in part to the historically low number of flu infections in the United States in the 2020-2021 season. As mask-wearing and social distancing became widespread during the COVID-19 pandemic, these public health measures also helped curb the spread of other airborne viruses, including among children.[49]

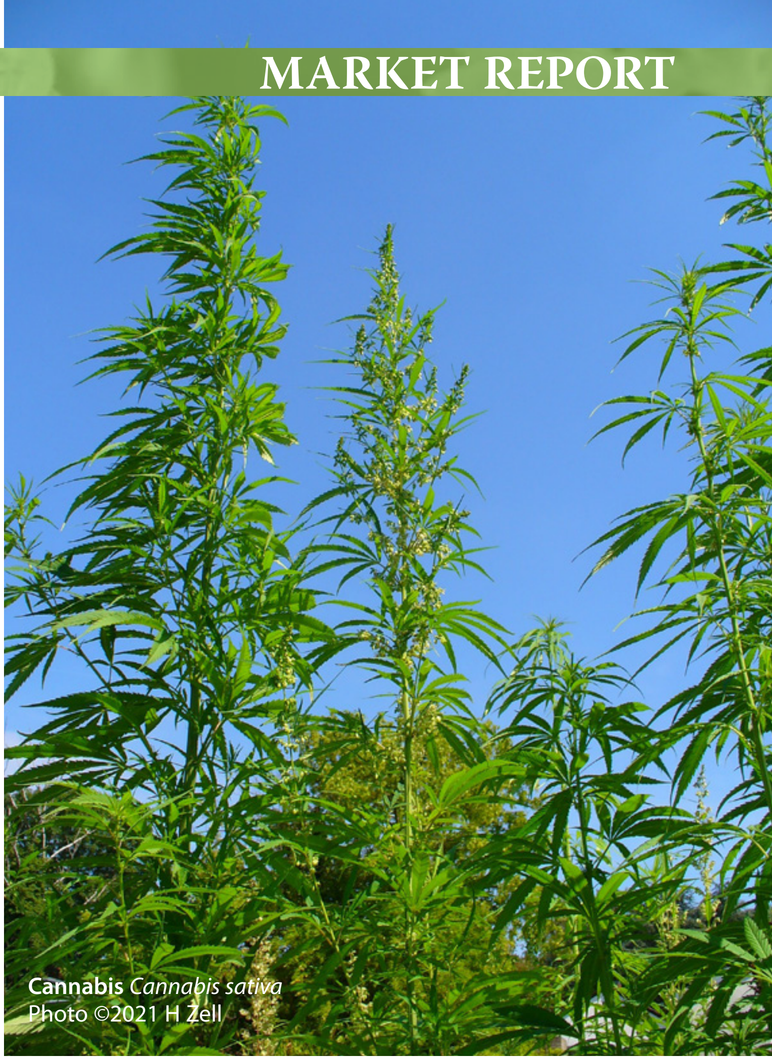
Cannabis *Cannabis sativa*
Photo ©2021 H Zell

Natural Channel Consumers Appear to Prioritize Immune and Cardiovascular Health Ingredients

CBD

For the third consecutive year, supplements with CBD as the primary ingredient had the highest total sales in the natural channel. Perhaps unexpectedly, CBD also had the largest sales decline of the top 40 natural channel ingredients. In 2020, consumers spent $57,217,025 on CBD supplements in natural retail outlets, a 36.9% decrease from the previous year. CBD was the only ingredient in the natural channel's top 40 list to have a sales decrease of more than 30%, and this is the first time CBD has seen a sales drop since it became one of the 40 top-selling supplements in this channel in 2017. Natural channel sales of CBD peaked in 2019, when consumers spent more than $90 million on these products — more than 11 times the annual sales of just two years earlier. Mainstream channel sales of CBD also decreased in 2020, with sales totaling $26,551,872, a 30% decrease from 2019.

In its CBD category, SPINS includes sales of products containing hemp-based CBD extracts, often marketed as

"full spectrum" extracts. The FDA defines hemp as *Cannabis sativa* with a tetrahydrocannabinol (THC, the primary psychoactive compound in cannabis) concentration of no more than 0.3%. *Cannabis sativa* with a THC concentration above 0.3% is legally classified as "cannabis" or "marijuana."[50]

Given CBD's widespread availability, from specialty health food stores to gas stations, consumers may think that such products are legal. However, this is technically not the case at the federal level. According to the FDA, "Even if a CBD product meets the definition of 'hemp' under the 2018 Farm Bill, it still must comply with all other applicable laws, including the FD&C Act [Federal Food, Drug, and Cosmetic Act]."[50] Under the FD&C Act, CBD and THC do not meet the criteria for legal dietary supplement ingredients. The FDA's primary CBD-related enforcement actions so far seem to consist largely of issuing warning letters. In 2020, the FDA sent 21 warning letters regarding CBD products, including 13 for unauthorized claims related to COVID-19.[51]

In addition to the potential regulatory confusion, the COVID-19 pandemic abruptly shifted consumers' purchasing habits and priorities, which may have also adversely impacted CBD sales. For most of 2020, many consumers were isolating at home and, when possible, minimizing trips in public and taking other measures to reduce risk of exposure to the virus. When consumers were able to visit natural retail outlets, it appears that many prioritized products with potential immune health benefits.[52]

Several negative media reports of CBD quality-control issues may have also contributed to decreased sales. A 2020 FDA analysis of 147 products advertised as containing CBD found that nine products contained no CBD, 64 contained less than the stated amount, 38 had more than the stated amount of CBD, and 72 contained more than the legally permitted amount of THC.[53] In May 2020, researchers analyzed the CBD content of 25 commercial CBD products purchased in Mississippi and found that only three (12%) contained within 20% of the amount stated on the label.[54]

ACV

ACV, as discussed previously, had the highest sales increase in the natural channel and was new to the top 40 natural channel list. Sales totaled $3,609,865 in 2020, a 97.7% increase from 2019. According to SPINS, the top health focuses for ACV supplements in the natural channel were digestive health, weight loss, pain and inflammation, and cold and flu.

Table 6. Total US Retail Sales of Herbal Supplements by Product Type (Single vs. Combination)

	Total Sales (in Billions)	% Total Sales	% Growth*
**2020**			
Single Herbs	$6.022	53.5%	11.5%
Combination Herbs	$5.238	46.5%	24.7%
**2019**			
Single Herbs	$5.402	56.3%	6.3%
Combination Herbs	$4.201	43.7%	11.8%
**2018**			
Single Herbs	$5.083	57.5%	6.8%
Combination Herbs	$3.759	42.5%	13.1%
**2017**			
Single Herbs	$4.759	58.9%	5.6%
Combination Herbs	$3.326	41.1%	12.9%
**2016**			
Single Herbs	$4.505	60.5%	6.1%
Combination Herbs	$2.947	39.5%	10.1%
**2015**			
Single Herbs	$4.245	61.3%	5.5%
Combination Herbs	$2.677	38.7%	10.7%

Source: *Nutrition Business Journal*
* From previous year

Quercetin

Quercetin had the second-highest percent sales increase in the natural channel. Sales totaled $6,415,921 in this channel in 2020, a 74.1% increase from 2019. Quercetin ranked 19th in sales in 2020. When it first appeared on the natural channel's top 40 list in 2017, it ranked 26th.

Quercetin is a plant pigment known as a flavonol, a type of flavonoid. Quercetin can be found in apples, berries, onions (*Allium cepa*, Amaryllidaceae), tea (*Camellia sinensis*, Theaceae), and grapes (*Vitis vinifera*, Vitaceae), among many other plants.[55] Quercetin has antioxidant and anti-inflammatory properties and has been used to treat many conditions, including allergies, heart disease, high cholesterol and blood pressure, and prostate issues such as BPH and prostate cancer.[56]

According to SPINS, natural channel consumers in 2020 spent the most on quercetin supplements marketed for allergies and respiratory health, followed by cardiovascular health and prostate support. Quercetin supplements for cardiovascular health and prostate support in particular experienced significant overall sales increases of 233.1% and 132.2%, respectively, in 2020. Quercetin supplements marketed for immune health, which make up a minority of quercetin supplement sales, also saw a sales increase of 59.2% in 2020.

At least two systematic reviews published in 2020 focused on the potential cardiovascular

benefits of quercetin. In one review of 17 human clinical trials, the authors concluded that quercetin supplementation was associated with significant reductions in systolic and diastolic blood pressure, and in a subgroup analysis of those who consumed quercetin for more than eight weeks, quercetin was associated with significantly improved levels of high-density lipoprotein (HDL) cholesterol ("good cholesterol") and triglycerides.[57] A separate review of 16 human clinical trials on quercetin supplementation in patients with metabolic syndrome and related conditions concluded that quercetin was associated with reduced levels of total cholesterol, LDL cholesterol, and C-reactive protein (a marker of inflammation associated with cardiovascular disease risk).[58]

Cardiovascular health, after "overall health and wellness" and immune health, was one of the most-cited reasons why dietary supplement users purchased such products in 2020, according to CRN's annual survey.[5] Maintaining cardiovascular health may have taken on renewed importance for some consumers in 2020 after the CDC listed pre-existing heart conditions, such as high blood pressure and coronary artery disease, as risk factors for developing severe illness from COVID-19 infection.[59,60] In June 2020, the CDC released data showing that patients with conditions such as cardiovascular disease (CVD) who contracted COVID-19 were "six times more likely to be hospitalized and 12 times more likely to die than patients without any chronic health problems." At that time, CVD was the most common underlying condition in patients with COVID-19.[61]

Several clinical trials and epidemiological studies have assessed the effects of quercetin on prostate conditions, such as BPH, prostatitis (inflammation of the prostate), and prostate cancer, with mixed results. In one randomized, controlled trial (RCT), supplementation with quercetin and green tea extract was not associated with changes in methylation levels in patients with prostate cancer.[62] (DNA methylation is involved in healthy gene expression.) A separate RCT published in 2012 found that quercetin may be effective for treating BPH.[63,64] Finally, a 2015 case-control study of men with prostate cancer reported a 27% reduction in the risk for prostate cancer for those who consumed at least 24 micrograms of quercetin daily.[65]

Four other ingredients in the natural channel saw sales increases greater than 30% in 2020: elder berry (68.2%), chaga (*Inonotus obliquus*, Hymenochaetaceae; 54.9%), oregano (*Origanum vulgare*, Lamiaceae; 43.2%), and mushrooms (other) (41.8%). Increased sales of elder berry, as discussed earlier, likely were boosted by the COVID-19 pandemic, as were sales of oregano supplements (including products labeled as containing oil of oregano and oregano leaf tinctures), which are commonly marketed for immune health.[66]

Mushroom Supplements

Consumers spent $2,730,506 on chaga mushroom supplements in natural retail stores in 2020, earning it a first-time spot in the top 40 list in this channel. Chaga fungi are amorphous tree parasites that live on various species, including birch (*Betula* spp., Betulaceae), alder (*Alnus* spp., Betulaceae), and beech (*Fagus* spp., Fagaceae).[67] For centuries, chaga preparations have been used traditionally in Russia, the Baltic states, China, Korea, and Japan for gastrointestinal issues, viral infections, heart health, and to stimulate the immune system.[68] Sales of chaga supplements, particularly powders, marketed for their antioxidant content and immune benefits, likely benefited from consumers' increased focus on immune health in 2020. Chaga has been called the "king of the medicinal mushrooms,"[69] but, despite this royal reputation, there were no known human clinical studies published as of July 2021.[67,70]

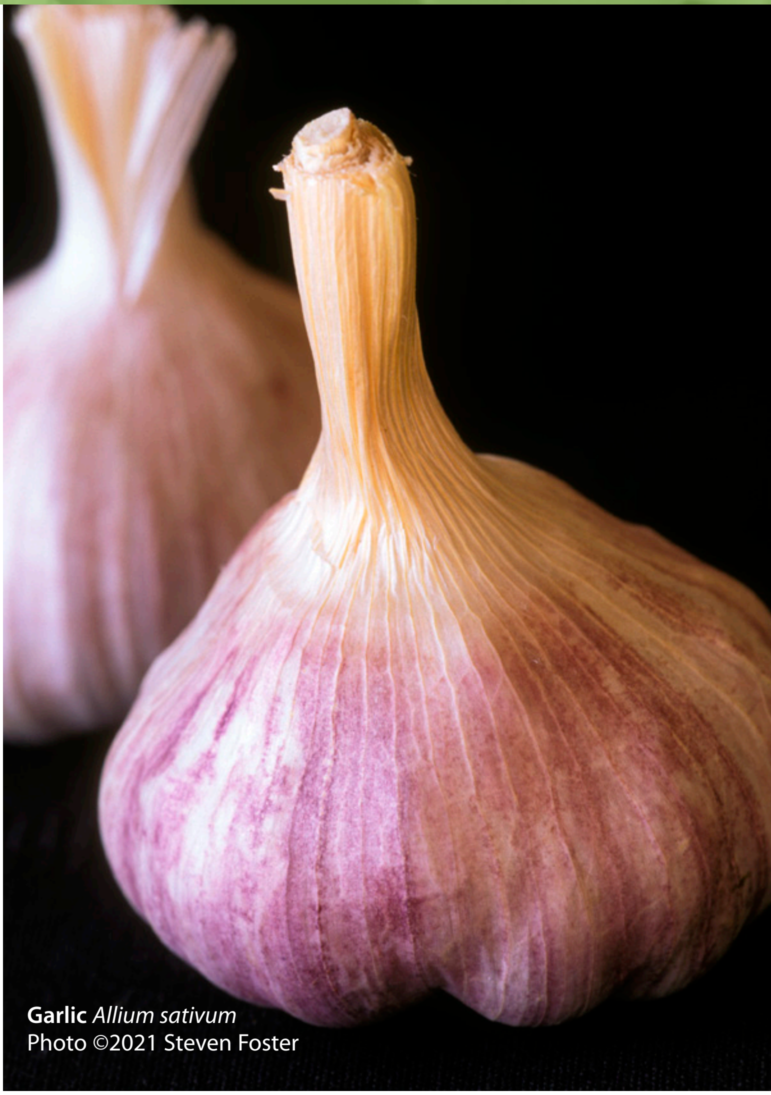

Garlic *Allium sativum*
Photo ©2021 Steven Foster

"Mushrooms (other)" is a category that includes sales of multiple fungal species not individually tracked by SPINS, including lion's mane (*Hericium erinaceus*, Hericiaceae), turkey tail (*Trametes versicolor*, Polyporaceae), and various combinations thereof. Natural channel sales in this category totaled $14,986,621 in 2020. Mushrooms (other) broke onto the natural channel scene in 2015, and since then, annual sales have more than tripled. From 2015 to 2020, mushrooms (other) moved from the 23rd top-selling supplement in this channel to the fifth.

According to SPINS, the top health focuses of mushrooms (other) in the 2020 natural channel were immune health and cognitive health, followed by energy support and mood support. Immune health and cognitive health have been the top health focuses of these supplements since 2018. In 2018 and 2019, the third top health focus was "non-specific uses" (e.g., general health benefits), but in 2020, total sales more than doubled for mushroom products marketed for energy and mood support.

Fungal supplements performed well overall in 2020. Of the 20 ingredients in the natural channel that saw sales increases in 2020, 20% were mushrooms: chaga, mushrooms (other), cordyceps (*Cordyceps* spp., Cordycipitaceae), and reishi (*Ganoderma lucidum*, Ganodermataceae). The so-called "shroom boom" of 2019 seems to have continued in 2020.[71]

Direct Sales

From 2017 to 2019, direct-to-consumer sales of herbal supplements experienced the highest percentage sales growth of NBJ's three market channels. However, in 2020, sales in the mass market channel had a higher percentage sales increase from 2019. Still, total direct sales of herbal supplements in 2020 were greater than both the mass market and natural, health food, and specialty channels combined. (Total annual direct sales of herbal supplements have been higher than total sales in the mass market channel and natural, health food, and specialty channel since at least 2005.)

The percentage sales growth in the direct channel was also significantly greater than in 2019. Sales in the direct channel grew by 23.7% in 2020, compared to 11.5% in 2019. NBJ tracks direct sales from four primary segments: multilevel marketing (MLM) companies, also known as network marketing companies, direct media (TV, radio, and print), health practitioners, and the internet.[47] According to NBJ, internet sales include e-commerce sales from Amazon.com and the websites of major brick-and-mortar retailers such as Walmart, among others.

Single-Herb Supplements vs. Combination Formulas

Since 2011, sales growth of combination formulas has been higher than that of single-herb supplements. This trend continued in 2020. Combination formula and single-herb supplement sales grew by 24.7% and 11.5%, respectively, from 2019 to 2020.

As noted in last year's *HerbalGram* Herb Market Report,[47] combination formulas contain multiple herbs that typically work together (additively or synergistically), often to support a general health function or body system. For example, a combination supplement marketed as a sleep aid may contain herbs or fungi with calming or anxiety-reducing properties and other ingredients with sleep-promoting effects.[72] Single-herb supplements, on the other hand, tend to have more targeted uses, although sometimes more than one. Senna (*Senna alexandrina*, Fabaceae), for example, has well-known laxative effects and is primarily used to treat digestive issues.[73] Although single-herb supplements have accounted for most of the total sales for more than a decade, the percentage gap between sales of these two product types has been slowly closing each year.

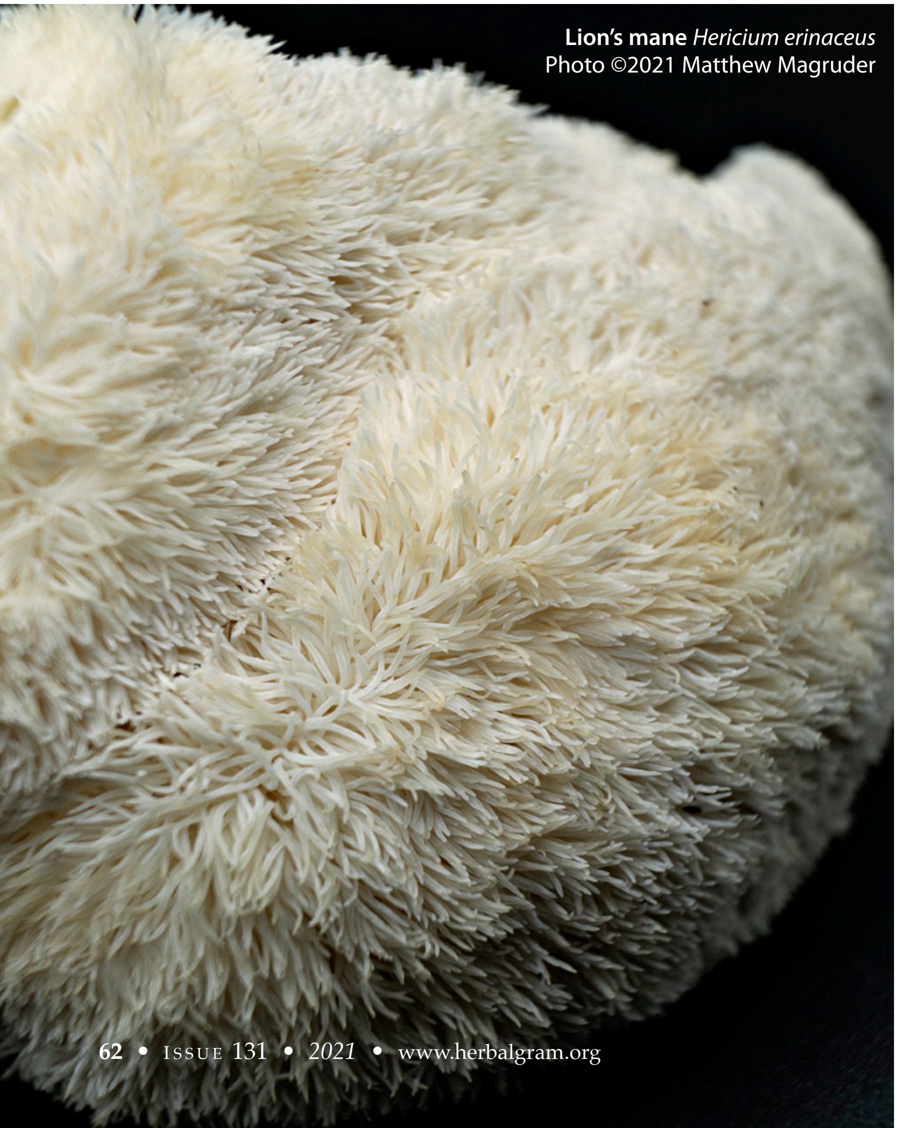

Lion's mane *Hericium erinaceus*
Photo ©2021 Matthew Magruder

Conclusion

In December 2020, *The Washington Post* asked readers to describe 2020 using a single word or phrase. The top three responses were revealing: chaotic, exhausting, lost. (Also in the top 10 were "nightmare," "relentless," and "dumpster fire," which Merriam-Webster defines as "an utterly calamitous or mismanaged situation or occurrence."[74,75]) According to the American Psychological Association's (APA's) *Stress in America 2020* report, those surveyed reported, among others, the pandemic, the US presidential election, and police violence toward minorities as significant

sources of stress in their lives, in addition to common, persistent stressors such as finances, work, and relationships. The APA warned of a coming second "pandemic," a mental health crisis, resulting from COVID-19-related stress.[76]

In this context, it is perhaps unsurprising that US consumers spent more than ever on herbal dietary supplements for immune health and stress relief in 2020. Herbs commonly used to support cardiovascular health, digestive health, and prostate health also performed well in 2020. This suggests that consumers may have been taking products not only to address immediate health concerns but also to ensure whole-body wellness in the midst of a global health crisis. 2020 may have been marked by chaos, exhaustion, and loss, but during a year in which much was out of control, many consumers seemed to take control of their own health and prioritized selfcare with herb- and fungi-based dietary supplements. As the pandemic stretches into its 20th month at the time of this writing (August 2021), it remains to be seen whether these trends and record-breaking sales will continue in 2021.

References

1. FDA 101: Dietary Supplements. US Food and Drug Administration website. Available at: www.fda.gov/consumers/consumer-updates/ fda-101-dietary-supplements. Accessed July 25, 2021.
2. Acimovic M, Jeremic K, Salaj N, et al. *Marrubium vulgare* L.: A phytochemical and pharmacological overview. *Molecules*. 2020;25(12):2898. Available at: www.mdpi.com/1420-3049/25/12/2898/htm. Accessed July 25, 2021.
3. Wieland LS, Piechotta V, Feinberg T, et al. Elderberry for prevention and treatment of viral respiratory illnesses: A systematic review. *BMC Complementary Medicine and Therapies*. 2021;21. Available at: https://bmccomplementmedtherapies.biomedcentral.com/articles/10.1186/s12906-021-03283-5. Accessed July 25, 2021.
4. Hawkins J, Baker C, Cherry L, Dunne E. Black elderberry (*Sambucus nigra*) supplementation effectively treats upper respiratory symptoms: A meta-analysis of randomized, controlled clinical trials. *Complementary Therapies in Medicine*. 2019;42:361-365. Available at: www.sciencedirect.com/ science/article/abs/pii/S0965229918310240. Accessed July 25, 2021.
5. 2020 CRN Survey Reveals Focus on Vitamins and Minerals. Council for Responsible Nutrition website. Available at: www.crnusa.org/newsroom/2020-crn-survey-reveals-focus-vitamins-and-minerals-available-purchase-consumer-survey. Accessed July 25, 2021.
6. AJMC Staff. A Timeline of COVID-19 Developments in 2020. *American Journal of Managed Care* website. Available at: www.ajmc.com/view/a-timeline-of-covid19-developments-in-2020. Accessed July 25, 2021.
7. Google Trends: Elderberry, 2020. Google Trends website. Available at: https://trends.google.com/trends/explore?date=2020-01-01%20 2020-12-31&geo=US&q=elderberry. Accessed July 25, 2021.
8. Health Information: Elderberry. National Center for Complementary and Integrative Health website. Available at: www.nccih.nih.gov/health/elderberry. Accessed July 25, 2021.
9. Fraudulent Coronavirus Disease 2019 (COVID-19) Products: Elderberry. US Food and Drug Administration website. Available at: www.fda.gov/consumers/health-fraud-scams/fraudulent-coronavirus-disease-2019-covid-19-products. Accessed July 25, 2021.
10. A comparison of Elderberry (*Sambucus nigra*) extract syrup and placebo against COVID-19 symptoms in outpatients and home quarantined patients: A randomized double-blind clinical trial. Iranian Registry of Clinical Trials website. Available at: https://en.irct.ir/trial/46958. Accessed July 25, 2021.
11. Help research treatments into Covid-19. June 24, 2021. East Kent Hospitals University/National Health Service Foundation Trust website. Available at: www.ekhuft.nhs.uk/patients-and-visitors/news-centre/latest-news/help-research-treatments-into-covid-19/. Accessed July 25, 2021.
12. 2020 CRN Consumer Survey on Dietary Supplements: Consumer Intelligence to Enhance Business Outcomes. Council for Responsible Nutrition website. Available at: www.crnusa.org/resources/2020-crn-consumer-survey-dietary-supplements-consumer-intelligence-enhance-business. Accessed July 25, 2021.
13. Singh N, Bhalla M, de Jager P, GIlca M. An overview on ashwagandha: A rasayana (rejuvenator) of Ayurveda. *Afr J Tradit Complement Altern Med*. 2011;8(S):208-213.
14. Panossian A, Wikman G. Effects of adaptogens on the central nervous system and the molecular mechanisms associated with their stress—protective activity. *Pharmaceuticals (Basel)*. 2010;3(1):188-224. Available at: www.ncbi.nlm.nih.gov/pmc/articles/PMC3991026/. Accessed July 25, 2021.
15. Panossian A, Wagner H. Adaptogens: A review of their history, biological activity, and clinical benefits. *HerbalGram*. 2011;90:52-63. Available at: http://herbalgram.org/resources/herbalgram/issues/90/table-of-contents/feat_adaptogens/. Accessed July 25, 2021.
16. Salve J, Pate S, Debnath K, Langade D. Adaptogenic and anxiolytic effects of ashwagandha root extract in healthy adults: A double-blind, randomized, placebo-controlled clinical study. *Cureus*. 2019;25;11(12):e6466. doi: 10.7759/cureus.6466. Available at: https://pubmed.ncbi.nlm.nih.gov/32021735/. Accessed July 25, 2021.
17. Bonilla DA, Moreno Y, Gho C, Petro JL, Odriozola-Martínez A, Kreider RB. Effects of ashwagandha (*Withania somnifera*) on physical performance: Systematic review and Bayesian meta-analysis. *Funct Morphol Kinesiol*. 2021;6(1):20. doi: 10.3390/jfmk6010020. Available at: https://pubmed.ncbi.nlm.nih.gov/33670194/. Accessed July 25, 2021.
18. Lopresti AL, Smith SJ, Drummond PD. Modulation of the hypothalamic-pituitary-adrenal (HPA) axis by plants and phytonutrients: a systematic review of human trials. *Nutr Neurosci*. 2021 Mar 2;1-27. doi: 10.1080/1028415X.2021.1892253. Available at: https://pubmed.ncbi.nlm.nih.gov/33650944/. Accessed July 25, 2021.
19. Aronson D. Cortisol — Its Role in Stress, Inflammation, and Indications for Diet Therapy. *Today's Dietician*. 2009;11(11):38. Available at: www.todaysdietitian.com/newarchives/111609p38.shtml. Accessed August 15, 2021.
20. CRN's COVID-19 Survey on Dietary Supplements: Consumer Insights on Usage and Attitudes about Dietary Supplements in Light of the Coronavirus Pandemic. Council for Responsible Nutrition website. Available at: www.crnusa.org/COVID19survey. Accessed July 25, 2021.
21. McDonald E. Debunking the health benefits of apple cider vinegar. August 23, 2018. University of Chicago Medicine website. Available at: https://www.uchicagomedicine.org/forefront/health-and-wellness-articles/debunking-the-health-benefits-of-apple-cider-vinegar. Accessed July 25, 2021.
22. Fahey RL. Health benefits of apple cider vinegar and other common vinegars: A review. *Integrative Medicine Alert*. 2017;20(6). Available at: www.proquest.com/openview/9db6b9a61f9a3f127ab3f13f39566c26/1?pq-origsite=gscholar&cbl=196259. Accessed July 25, 2021.
23. Hoover A. Apple Cider Vinegar Myths & Facts. West Virginia University Extension Service website. Available at: https://extension.wvu.edu/food-health/cooking/apple-cider-vinegar-myths-facts. Accessed July 25, 2021.
24. Taylor EJ. Alkaline or Acid? September 18, 1960. *The New York Times*. Available at: https://timesmachine.nytimes.com/timesmachine/1960/09/18/99872768.html?pageNumber=158. Accessed July 26, 2021.
25. Bauman H. Free fire cider: Traditional remedy remains generic after landmark case. *HerbalGram*. 2020;125:58-65. Available at: http://herbalgram.org/resources/herbalgram/issues/125/table-of-contents/hg125-feat-firecider/. Accessed July 25, 2021.
26. Straus M. Apple cider vinegar: The Science and the Trends. December 29, 2020. *Nutritional Outlook*. Available at: www.nutritionaloutlook.com/view/apple-cider-vinegar-the-science-and-the-trends. Accessed July 25, 2021.
27. Cassetty S. The Health Benefits of Apple Cider Vinegar, According to a Dietitian. July 22, 2020. Today website. Available at: www.today.com/health/benefits-apple-cider-vinegar-according-dietitian-t187438. Accessed July 25, 2021.
28. Google Trends: Apple cider vinegar, 2020. Google Trends website. Available at: https://trends.google.com/trends/explore?date=2020-01-01%202020-12-31&geo=US&q=apple%20cider%20vinegar. Accessed July 25, 2021.

29. Goli Nutrition Launches World's First Apple Cider Vinegar Gummy [press release]. West Hollywood, CA: Goli Nutrition; September 23, 2019. Available at www.prnewswire.com/news-releases/goli-nutrition-launches-worlds-first-apple-cider-vinegar-gummy-300922883.html. Accessed July 25, 2021.
30. Ewe K. Is Apple Cider Vinegar Really Good for You? I Put It to the Ultimate Test. April 27, 2021. VICE website. Available at: www.vice.com/en/article/88naqp/apple-cider-vinegar-health-beauty-benefits-test. Accessed July 25, 2021.
31. Cheng LJ, Jiang Y, Wu VX, Wang W. A systematic review and meta-analysis: Vinegar consumption on glycaemic control in adults with type 2 diabetes mellitus. *Journal of Advanced Nursing*. 2019;76(2):459-474. Available at: https://onlinelibrary.wiley.com/doi/10.1111/jan.14255. Accessed July 25, 2021.
32. Martini N. Apple cider vinegar. *Journal of Primary Health Care.* 2021;13(2):191-192. https://doi.org/10.1071/HC19561. Available at: www.publish.csiro.au/HC/fulltext/HC19561. Accessed July 25, 2021.
33. Hadi A, Pourmasoumi M, Najafgholizadeh A, Clark CCT, Esmaillzadeh A. The effect of apple cider vinegar on lipid profiles and glycemic parameters: A systematic review and meta-analysis of randomized clinical trials. *BMC Complement Med Ther*. 2021;21(1):179. doi: 10.1186/s12906-021-03351-w. Available at: https://pubmed.ncbi.nlm.nih.gov/34187442/. Accessed July 25, 2021.
34. Launholt TL, Kristiansen CB, Hjorth P. Safety and side effects of apple vinegar intake and its effect on metabolic parameters and body weight: a systematic review. *Eur J Nutr*. 2020;59(6):2273-2289. doi: 10.1007/s00394-020-02214-3. Available at: https://pubmed.ncbi.nlm.nih.gov/32170375/. Accessed July 25, 2021.
35. Higdon J, Drake VJ, Delage B. Micronutrient Information Center: Phytosterols. Oregon State University Linus Pauling Institute website. Available at: https://lpi.oregonstate.edu/mic/dietary-factors/phytochemicals/phytosterols. Accessed July 25, 2021.
36. Bin Sayeed MS, Karim SMR, Sharmin T, Morshed MM. Critical analysis on characterization, systemic effect, and therapeutic potential of beta-sitosterol: A plant-derived orphan phytosterol. *Medicines (Basel)*. 2016;3(4):29. Available at: www.ncbi.nlm.nih.gov/pmc/articles/PMC5456237/. Accessed July 25, 2021.
37. Beta-sitosterol plant extract. University of Michigan Health website. Available at: www.uofmhealth.org/health-library/ug1904spec. Accessed July 25, 2021.
38. Prostate Enlargement (Benign Prostatic Hyperplasia). National Institute of Diabetes and Digestive and Kidney Diseases website. Available at: www.niddk.nih.gov/health-information/urologic-diseases/prostate-problems/prostate-enlargement-benign-prostatic-hyperplasia#howCommon. Accessed July 25, 2021.
39. Wilt T, Ishani A, Stark G, Mulrow C, Lau J. Beta-sitosterols for benign prostatic hyperplasia. *Cochrane Database Syst Rev*. 2000;(2):CD001043. doi: 10.1002/14651858.CD001043. Available at: https://pubmed.ncbi.nlm.nih.gov/10796740/. Accessed July 25, 2021.
40. Parsons JK. Benign prostatic hyperplasia and male lower urinary tract symptoms: Epidemiology and risk factors. *Curr Bladder Dysfunct Rep*. 2010; 5(4): 212–218. Available at: www.ncbi.nlm.nih.gov/pmc/articles/PMC3061630/. Accessed July 25, 2021.
41. Ras RT, Gelejinse JM, Trautwein EA. LDL-cholesterol-lowering effect of plant sterols and stanols across different dose ranges: a meta-analysis of randomised controlled studies. *Br J Nutr*. 2014;112(2): 214–219. Available at: www.ncbi.nlm.nih.gov/pmc/articles/PMC4071994/. Accessed July 25, 2021.
42. Ghaedi E, Foshati S, Ziaei R, et al. Effects of phytosterols supplementation on blood pressure: A systematic review and meta-analysis. *Clinical Nutrition*. 2019;39(9):2702-2710. Available at: www.clinicalnutritionjournal.com/article/S0261-5614(19)33206-6/fulltext. Accessed July 25, 2021.
43. Straus M. Men's Healthy-Aging Supplements Market: New Ingredient Research, New Delivery Forms. September 4, 2019. *Nutritional Outlook*. Available at: www.nutritionaloutlook.com/view/mens-healthy-aging-supplements-market-new-ingredient-research-new-delivery-forms. Accessed July 25, 2021.
44. 65 and Older Population Grows Rapidly [press release]. Washington, DC: United States Census Bureau; June 25, 2020. Available at: www.census.gov/newsroom/press-releases/2020/65-older-population-grows.html. Accessed July 25, 2021.
45. Bodagh MN, Maleki I, Hekmatdoost Z. Ginger in gastrointestinal disorders: A systematic review of clinical trials. *Food Sci Nutr*. 2019;7(1):96-108. Available at: www.ncbi.nlm.nih.gov/pmc/articles/PMC6341159/. Accessed July 25, 2021.
46. Srivastava JK, Shankar E, Gupta S. Chamomile: A herbal medicine of the past with bright future. *Mol Med Report*. 2010;3(6):895-901. Available at: www.ncbi.nlm.nih.gov/pmc/articles/PMC2995283/. Accessed July 25, 2021.
47. Smith T, May G, Eckl V, Morton Reynolds C. US sales of herbal supplements increase by 8.6% in 2019: CBD, mushroom, and elderberry supplements continue to drive sales. *HerbalGram*. 2020;127:54-67. Available at: http://herbalgram.org/media/15608/hg127-hmr.pdf. Accessed July 25, 2021.
48. Sierocinski E, Holzinger F, Chernot JF. Ivy leaf (*Hedera helix*) for acute upper respiratory tract infections: an updated systematic review. *Eur J Clin Pharmacol*. 2021;77(8):1113-1122. Available at: www.ncbi.nlm.nih.gov/pmc/articles/PMC8275562/. Accessed July 25, 2021.
49. Rodriguez A. Flu Cases Were at an All-Time Low during the 2020-2021 Season. What Experts Say to Expect in Next Season's Vaccine. May 10, 2021. *USA Today*. Available at: www.usatoday.com/story/news/health/2021/05/10/flu-cases-historically-low-during-covid-what-expect-fall/7088318002/. Accessed July 25, 2021.
50. FDA Regulation of Cannabis and Cannabis-Derived Products, Including Cannabidiol (CBD). US Food & Drug Administration website. Available at: www.fda.gov/news-events/public-health-focus/fda-regulation-cannabis-and-cannabis-derived-products-including-cannabidiol-cbd. Accessed July 25, 2021.
51. Warning Letters and Test Results for Cannabidiol-Related Products. US Food and Drug Administration website. Available at: www.fda.gov/news-events/public-health-focus/warning-letters-and-test-results-cannabidiol-related-products. Accessed July 25, 2021.
52. Runestad T. Why Did the Hemp CBD Market Stagger through 2020? November 25, 2020. New Hope Network website. Available at: www.newhope.com/webinars-toolkits-and-downloads/why-did-hemp-cbd-market-stagger-through-2020. Accessed July 25, 2021.
53. Schultz H. FDA Report Details Poor Quality Control, Undisclosed THC in Many CBD Products. July 9, 2020. NutraIngredients-USA website. Available at: www.nutraingredients-usa.com/Article/2020/07/09/FDA-report-details-poor-quality-control-undisclosed-THC-in-many-CBD-products. Accessed July 25, 2021.
54. Gurley BJ, Murphy TP, Gul W, Walker LA, ElSohly M. Content versus label claims in cannabidiol (CBD)-containing products obtained from commercial outlets in the state of Mississippi. *Journal of Dietary Supplements*. 2020;17(5):599-607. Available at: www.tandfonline.com/doi/abs/10.1080/19390211.2020.1766634. Accessed July 25, 2021.
55. Li Y, Yao J, Han C, et al. Quercetin, inflammation and immunity. *Nutrients*. 2016;8(167). doi:10.3390/nu8030167. Available at: www.ncbi.nlm.nih.gov/pmc/articles/PMC4808895/pdf/nutrients-08-00167.pdf. Accessed July 25, 2021.
56. Health Library: Quercetin. Icahn School of Medicine at Mount Sinai website. Available at: www.mountsinai.org/health-library/supplement/quercetin. Accessed July 25, 2021.
57. Huang H, Liao D, Dong Y, Pu R. Effect of quercetin supplementation on plasma lipid profiles, blood pressure, and glucose levels: a systematic review and meta-analysis. *Nutr Rev*. 2020;78(8):615-626. Available at: https://pubmed.ncbi.nlm.nih.gov/31940027/. Accessed July 25, 2021.
58. Tabrizi R, Tamtaji OR, Mirhosseini N, et al. The effects of quercetin supplementation on lipid profiles and inflammatory markers among patients with metabolic syndrome and related disorders: A systematic review and meta-analysis of randomized controlled trials. *Crit Rev Food Sci Nutr*. 2020;60(11):1855-1868. Available at: https://pubmed.ncbi.nlm.nih.gov/31017459/. Accessed July 25, 2021.
59. Science Brief: Evidence Used to Update the List of Underlying Medical Conditions that Increase a Person's Risk of Severe Illness from COVID-19. Centers for Disease Control and Prevention website. Available at: www.cdc.gov/coronavirus/2019-ncov/science/science-briefs/underlying-evidence-table.html. Accessed July 25, 2021.
60. Sloan AE, Hutt CA. Getting Ahead of the Curve: COVID Refocuses Attention on Heart Health. October 1, 2020. *Nutraceuticals World*. Available at: www.nutraceuticalsworld.com/issues/2020-10/view_trendsense/getting-ahead-of-the-curve-covid-refocuses-attention-on-heart-health/1032. Accessed July 25, 2021.

61. Morbidity and Mortality Weekly Report: Coronavirus Disease 2019 Case Surveillance. Centers for Disease Control and Prevention website. Available at: www.cdc.gov/mmwr/volumes/69/wr/mm6924e2.htm?s_cid=mm6924e2_w. Accessed July 25, 2021.
62. Ghafouri-Fard S, Shabestari FA, Vaezi S, et al. Emerging impact of quercetin in the treatment of prostate cancer. *Biomedicine & Pharmacotherapy*. 2021;138:111548. Available at: www.sciencedirect.com/science/article/pii/S0753332221003334. Accessed July 25, 2021.
63. Ghorbanibirgani A. Efficacy of quercetin in treatment of benign prostatic hyperplasia in a double-blind randomized clinical trial in Iran-2011. *Contraception*. 2012;85:321. Available at: www.ncbi.nlm.nih.gov/pmc/articles/PMC5408066/#B30. Accessed July 25, 2021.
64. Eleazu C, Eleazu K, Kalu W. Management of benign prostatic hyperplasia: Could dietary polyphenols be an alternative to existing therapies? *Front Pharmacol*. 2017;8:234. Available at: www.ncbi.nlm.nih.gov/pmc/articles/PMC5408066/#B30. Accessed July 25, 2021.
65. Yang F, Song L, Wang H, Wang J, Xu Z, Xing N. Quercetin in prostate cancer: Chemotherapeutic and chemopreventive effects, mechanisms and clinical application potential (Review). *Oncology Reports*. 2015;33(6):2659-2668. Available at: www.spandidos-publications.com/or/33/6/2659#b19-or-33-06-2659. Accessed July 25, 2021.
66. Garnier A, Shahidi F. Spices and herbs as immune enhancers and anti-inflammatory agents: A review. *J Food Bioact*. 2021;14:20-52. Available at: www.isnff-jfb.com/index.php/JFB/article/view/225/363. Accessed July 27, 2021.
67. Szychowski KA, Skora B, Pomianek T, Gminski J. *Inonotus obliquus* — from folk medicine to clinical use. *J Tradit Complement Med*. 2021;11(4):293-302. Available at: www.ncbi.nlm.nih.gov/pmc/articles/PMC8240111/. Accessed July 25, 2021.
68. Gery A, Dubreule C, Andre V, et al. Chaga (*Inonotus obliquus*), a future potential medicinal fungus in oncology? A chemical study and a comparison of the cytotoxicity against human lung adenocarcinoma cells (A549) and human bronchial epithelial cells (BEAS-2B). *Integr Cancer Ther*. 2018;17(3):832-843. Available at: www.ncbi.nlm.nih.gov/pmc/articles/PMC6142110/#bibr13-1534735418757912. Accessed July 25, 2021.
69. Wolfe D. *Chaga: King of the Medicinal Mushrooms*. Berkeley, CA: North Atlantic Books; 2012.
70. "Inonotus obliquus," Clinical Trial [search]. PubMed website. Available at: https://pubmed.ncbi.nlm.nih.gov/?term=Inonotus+obliquus&filter=pubt.clinicaltrial&sort=date. Accessed July 27, 2021.
71. Berry S. Australians Are Riding the 'Shroom Boom.' April 24, 2019. *The Sydney Morning Herald*. Available at: www.smh.com.au/lifestyle/health-and-wellness/australians-are-riding-the-shroom-boom-20190423-p51gfo.html. Accessed July 25, 2021.
72. Horne S. Understanding Herbal Formulas. American Herbalists Guild website. Available at: www.americanherbalistsguild.com/sites/default/files/Proceedings/horne_steven_-_understanding_herbal_formulas.pdf. Accessed July 25, 2021.
73. Brinckmann J, Smith T. Senna: *Cassia angustifolia* and *Cassia senna* (syn. *Cassia acutifolia*, *Senna alexandrina*) Family: Fabaceae (Leguminosae). *HerbalGram*. 2018;120:6-13. Available at: https://www.herbalgram.org/resources/herbalgram/issues/120/table-of-contents/hg120-herbpro-senna/. Accessed August 4, 2021.
74. Goren E, Kulkarni SS, Vongkiatkajorn K. *The Washington Post* Asked Readers to Describe 2020 in One Word or Phrase. Here's What They Said. December 18, 2020. *The Washington Post*. Available at: www.washingtonpost.com/graphics/2020/lifestyle/2020-in-one-word/. Accessed July 25, 2021.
75. Dumpster fire. Merriam-Webster website. Available at: www.merriam-webster.com/dictionary/dumpster%20fire. Accessed July 25, 2021.
76. Stress in America 2020: A National Mental Health Crisis. Washington, DC: American Psychological Association; October 2020. Available at: www.apa.org/news/press/releases/stress/2020/sia-mental-health-crisis.pdf. Accessed July 25, 2021.

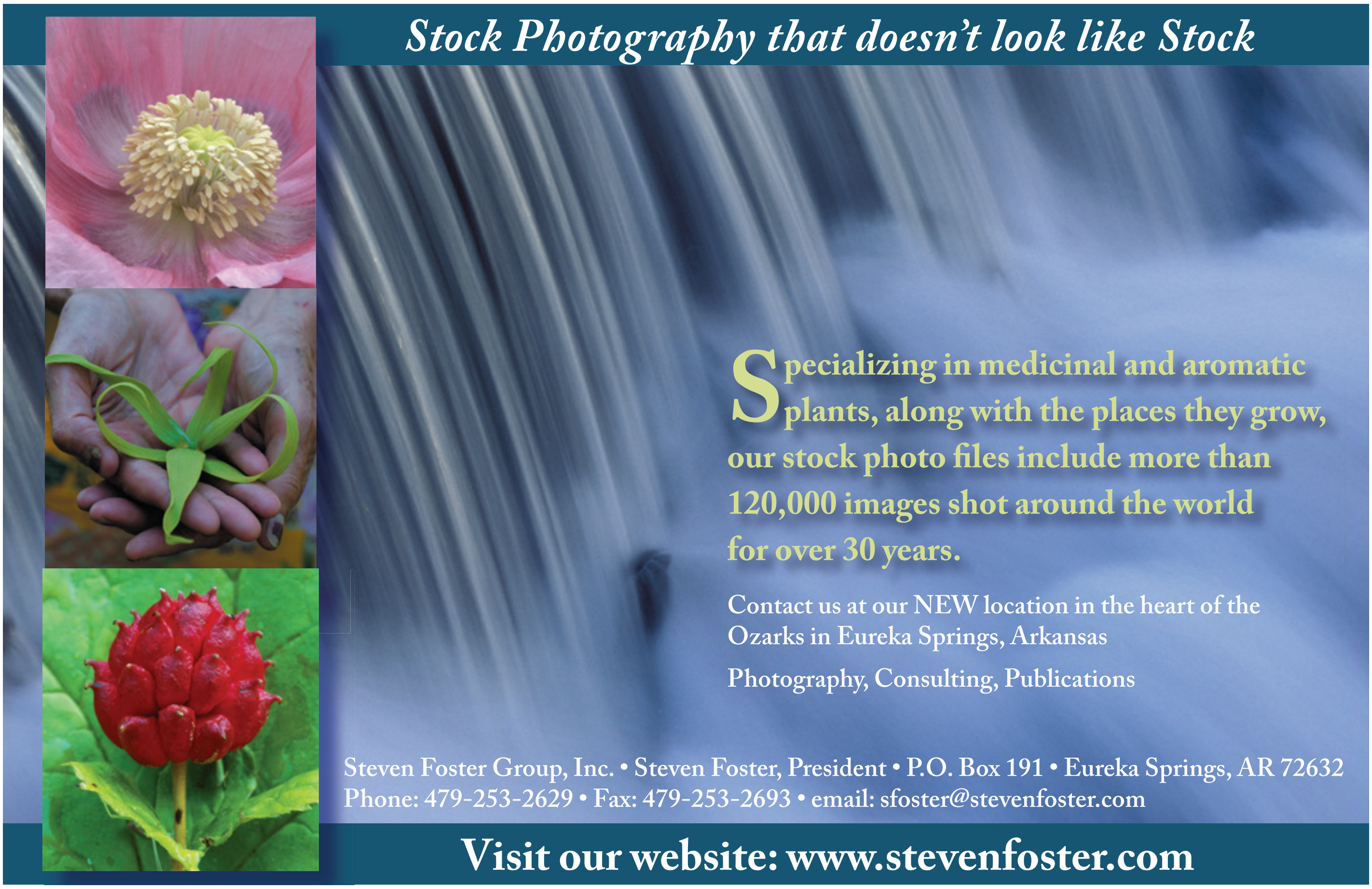

Supplement: Supplementary file 2 — Supplementary file2 (PDF 2134 KB) [file 280_2023_4504_MOESM2_ESM.pdf]
